# Biosourced quinones for high-performance environmentally benign electrochemical capacitors via interface engineering

**DOI:** 10.1038/s42004-022-00719-y

**Published:** 2022-08-20

**Authors:** Abdelaziz Gouda, Alexandre Masson, Molood Hoseinizadeh, Francesca Soavi, Clara Santato

**Affiliations:** 1 Department of Engineering Physics, Polytechnique Montreal, C.P. 6079, Succ. Centre-ville, Montreal, Quebec H3C 3A7 Canada; 2 Department of Chemistry “Giacomo Ciamician”, Alma Mater Studiorum Università di Bologna, Via Selmi, 2, Bologna, 40126 Italy; 3 Present Address: Now at, Solar Fuels Research Group, Department of Chemistry, University of Toronto, 80 St. George Street, Toronto, M5S 3H6 Canada

**Keywords:** Supercapacitors, Bioinspired materials

## Abstract

Biosourced and biodegradable organic electrode materials respond to the need for sustainable storage of renewable energy. Here, we report on electrochemical capacitors based on electrodes made up of quinones, such as *Sepia* melanin and catechin/tannic acid (Ctn/TA), solution-deposited on carbon paper engineered to create high-performance interfaces. *Sepia* melanin and Ctn/TA on TCP electrodes exhibit a capacitance as high as 1355 mF cm^−2^ (452 F g^−1^) and 898 mF cm^−2^ (300 F g^−1^), respectively. *Sepia* melanin and Ctn/TA symmetric electrochemical capacitors operating in aqueous electrolytes exhibit up to 100% capacitance retention and 100% coulombic efficiency over 50,000 and 10,000 cycles at 150 mA cm^−2^ (10 A g^−1^), respectively. Maximum power densities as high as 1274 mW cm^−2^ (46 kW kg^−1^) and 727 mW cm^−2^ (26 kW kg^−1^) with maximum energy densities of 0.56 mWh cm^−2^ (20 Wh kg^−1^) and 0.65 mWh cm^−2^ (23 Wh kg^−1^) are obtained for Sepia melanin and Ctn/TA.

## Abstract (web summary)

Biosourced and biodegradable organic electrode materials are investigated for environmentally benign energy storage, but their performance at higher current density is often poor. Here, the authors construct electrodes with quinone-based species from Sepia melanin and tannins on treated carbon paper and observe electrode capacitance as high as 1355 mF cm^−2^ at current densities up to 10 A g^−1^.

## Introduction

Environmental concerns related to global warming necessitate migration from fossil fuel energy to renewable energy. However, the most promising renewable energy sources, sun and wind, are intermittent and dependent on predictable but uncontrollable meteorological phenomena^1^. Thus, the migration in energy sources has to be accompanied by the development of electrical grids and/or energy storage facilities. In addition, the increased use of electric vehicles and portable electronic devices and the development of the Internet of Things require low-cost and sustainable power sources^2,3^.

Energy storage is recognized as the key technology for a decarbonized economy by the European Commission Energy Roadmap 2050^4^. Commercially available electrochemical energy storage devices often make use of electrode materials that are produced by processes or include materials that are costly and have dramatic environmental impacts^1,5–7^. The recent Batteries Europe Strategic Research Agenda stresses that “future research and development activities on batteries must address environmental sustainability by developing methodologies and technologies to optimize battery production, minimize resource and energy use, and strive to achieve the lowest possible environmental footprint of batteries”^8^.

Despite having about ten times less energy density than batteries, supercapacitors can deliver hundred times more power density and perform thousand times more charge-discharge high-rate cycles than batteries. They are rapidly recharged and find applications where peak power is required: ignition systems, emergency doors in aircrafts, power grids to improve the lifespan of storage systems by smoothening power fluctuations, regenerative braking in vehicles, wearable electronics, space applications, and in vivo medical devices^9–12^.

Electrochemical double layer capacitors (EDLCs) are the most common electrochemical capacitors (supercapacitors). Carbon is widely used for EDLCs for the abundance, low cost, high surface area, and conductivity of some of its forms^13^. EDLCs store/deliver charge by a rapid electrostatic process. The charge storage capability of carbon can be increased in pseudosupercapacitors by depositing redox-active materials that undergo fast and reversible Faradic processes. As EDLCs, pseudosupercapacitor electrodes provide box-shaped voltammetries and triangular galvanostatic charge/discharge profiles (as opposed to battery-like electrodes that feature voltammetric peaks and galvanostatic charge/discharge profile plateaus)^14–19^.

The redox activity of quinone-based molecules, such as melanins, lignin, and tannins, permits higher charge storage performance through pseudocapacitance in supercapacitors. Quinone and quinone derivatives have been exploited to enhance the charge storage capacity of carbon electrodes^20–23^. In aqueous solutions, quinones undergo two-electron, proton-coupled electron transfers^24–29^. Biosourced, quinone-based organic electrode materials operating in aqueous electrolytes represent a promising option for next-generation sustainable energy storage devices (Supplementary Table 1, portion on biosourced quinone-based materials). Polyanthraquinone/carbon electrodes in 0.5 M LiCl_4_ in acetonitrile exhibit specific capacitance of up to 650 F g^−1^ with 88% capacitance retention over 1000 cycles; corresponding asymmetric supercapacitors making use of a second electrode based on graphene feature energy density up to 45.5 Wh kg^−1^ and power density up to 21.4 kW kg^−1^^20^. Electrodes based on polydopamine on functionalized carbon cloth feature specific capacitance of 617 mF cm^−2^ (626 F g^−1^) in PVA-H_2_SO_4_ electrolyte, with cycling stability of 81% over 10,000 cycles; symmetric supercapacitors built from these electrodes exhibit maximum energy and power density of 11.7 Wh kg^−1^ and 6.4 kW kg^−1^, respectively^30^. Carbonized chitosan-amino acid gel supercapacitors show maximum specific capacitance of ca. 478 F g^−1^ in 6 M KOH with 100% cycling stability after 100,000 cycles, and 30 Wh kg^−1^ and 225 W kg^−1^ maximum energy and power density, respectively^31^. Pyrolyzed benzoquinone-amine supercapacitors show maximum specific capacitance of ca. 360 F g^−1^ in 1 M H_2_SO_4_ with 90% cycling stability after 100 000 cycles, and 18.2 Wh kg^−1^ and 300 W kg^−1^ maximum energy and power density, respectively^22^. Perylene diimide and hexaazatrinaphthylene (PHATN)-based electrodes exhibit a specific capacitance of 689 F g^−1^ in 6 M KOH. An asymmetric electrochemical capacitor from PHATN and activated carbon shows 100% Coulombic efficiency and 50% capacity retention after 10 000 cycles at 20 A g^−1^^16^.

Unfortunately, issues such as high contact resistance at the quinone/carbon interface in pseudosupercapacitors lead to poor rate response (loss in performance at higher current densities) and short cycling stability, thus hindering the commercial development of the devices^27,29,30,32^. Therefore, the engineering of such interfaces is deemed imperative.

Eumelanin is a quinone-based biomacromolecule belonging to the melanin family. *Sepia* melanin (indicated as *Sepia* melanin or sepia from here on) is a natural eumelanin extracted from the ink sac of cuttlefish (*Sepia officinalis*)^33,34^. It features fascinating properties such as redox activity, strong broadband UV-visible absorption, metal-binding affinity, hydration-dependent electrical response, possible electronic transport, and good thermal and photo stability^35,36^. Our group investigated the biodegradability in industrial compost conditions of eumelanin for sustainable (green) organic electronics and their powering elements^37^.

**Fig. 1:**
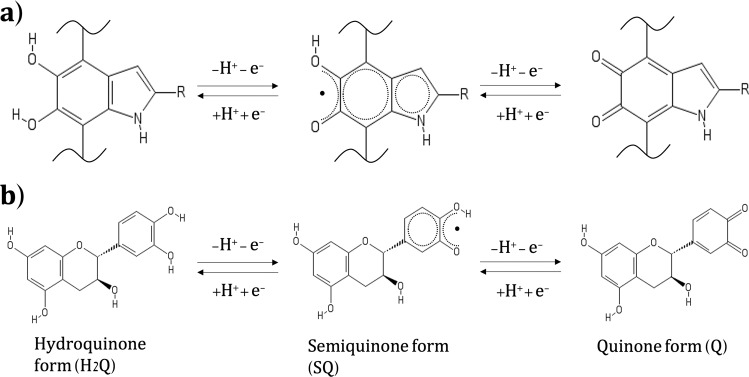
Schematic representation of quinone redox forms. **a** Redox forms of the building blocks of eumelanin: 5,6-dihydroxyindole (DHI) and 5,6 dihydroxyindole-2-carboxylic acid (DHICA). R is −H in DHI and −COOH in DHICA. **b** Redox forms from catechol to catequinone for catechin molecule.

Eumelanin is made of two main building blocks, 5,6-dihydroxyindole (DHI) and 5,6-dihydroxyindole-2-carboxylic acid (DHICA), coexisting in different redox states (Fig. 1a). The redox activity of eumelanin combined with its capability to reversibly bind multivalent cations constitute the foundation for the use of eumelanin in energy storage systems^29,38^. Eumelanin-based electrodes have been reported for flexible micro supercapacitors, light-assisted supercapacitors, and secondary Na^+^ and Mg^2+^ batteries^28,29,39–43^. We studied eumelanin aqueous supercapacitors operating at different pH values^29,39,40^. Our studies reported relatively low specific capacitance values (up to 5.6 mF cm^−2^), attributable to the low electronic conductivity of eumelanin and high contact resistance at the eumelanin/current collector interface.

Tannins can easily be extracted from a wide range of natural precursors^27,44–48^. Tannins have been used since antiquity in leather treatment and wine production^27,49,50^. A wide variety of tannins, particularly tannic acid (TA), have been used as electrode materials for supercapacitors^51^ and cathodes for lithium-ion batteries^52^, owing to their redox properties (reversible oxidation of the catechol group into a quinone, Fig. 1b). TA has also been used to improve the capacitance of polydopamine-coated electrodes, using the interactions between Fe^3+^ and TA to create strong and durable metal-phenol bonds for flexible carbon-based supercapacitors^53^. Moreover, the hydrogen-bonding capability of TA has been exploited to use it as a small-molecule binder to improve the stability of silicon anodes in Li-ion batteries^54^ and also to enhance the mechanical strength of carbon nanotubes (CNT) and reduced graphene oxide (r-GO) electrodes for flexible supercapacitor applications^51,55^.

The great diversity of the tannin family is a double-edged sword, on the one hand giving a wide range of candidate molecules and sources but on the other making it more difficult to find the optimal solution for supercapacitor applications. Catechin (Ctn) is a member of this family as a part of the condensed tannins branch (macromolecules composed of smaller phenolic components)^49^. It has been reported that the use of hydrolysable chestnut bark tannins and polypyrrole (ppy) greatly increases the capacitance (from 100 F g^−1^ for ppy alone to 370 F g^−1^ for the ppy-tannin composite) of a carbonized wood electrode through simple galvanostatic deposition of tannins and ppy in aqueous solution^27^.

Here, we report on environmentally friendly and high-performance pseudosupercapacitors based on chemically engineered carbon modified by solution-processing with two quinone materials, namely *Sepia* melanin and catechin, and operating in a mild aqueous electrolyte. Brunauer-Emmett-Teller (BET) surface area measurements, scanning electron microscopy (SEM), X-ray diffraction (XRD), Raman spectroscopy, and X-ray photoelectron spectroscopy (XPS) were used to investigate the surface area, morphology, and chemistry of the electrode materials. Cyclic voltammetry, galvanostatic charge/discharge, and electrochemical impedance spectroscopy were performed to study the electrochemical behavior of the electrodes and characterize the performance of supercapacitors based thereon.

## Results and discussion

A key component in our strategy for environmentally benign, high-performance energy storage is the engineering of the quinone/carbon interfaces through the modification of the carbon surface.

### Morphological and chemical characterization

#### CP and TCP

We treated carbon paper (CP) using a two-step chemical method (18 M H_2_SO_4_/16 M HNO_3_ (3:1 v/v)) and 7 M (NH_4_)_2_HPO_4_ salt, in controlled temperature conditions^19,56^.

SEM was used to investigate the surface morphology of CP, treated carbon paper (TCP) and biosourced quinones (sepia or catechin) on TCP. SEM images of CP and TCP show surface grooves (Fig. 1a, b); long whiskers are observable for TCP, imparting a relatively coarse surface for better biosourced material hosting.

**Fig. 2:**
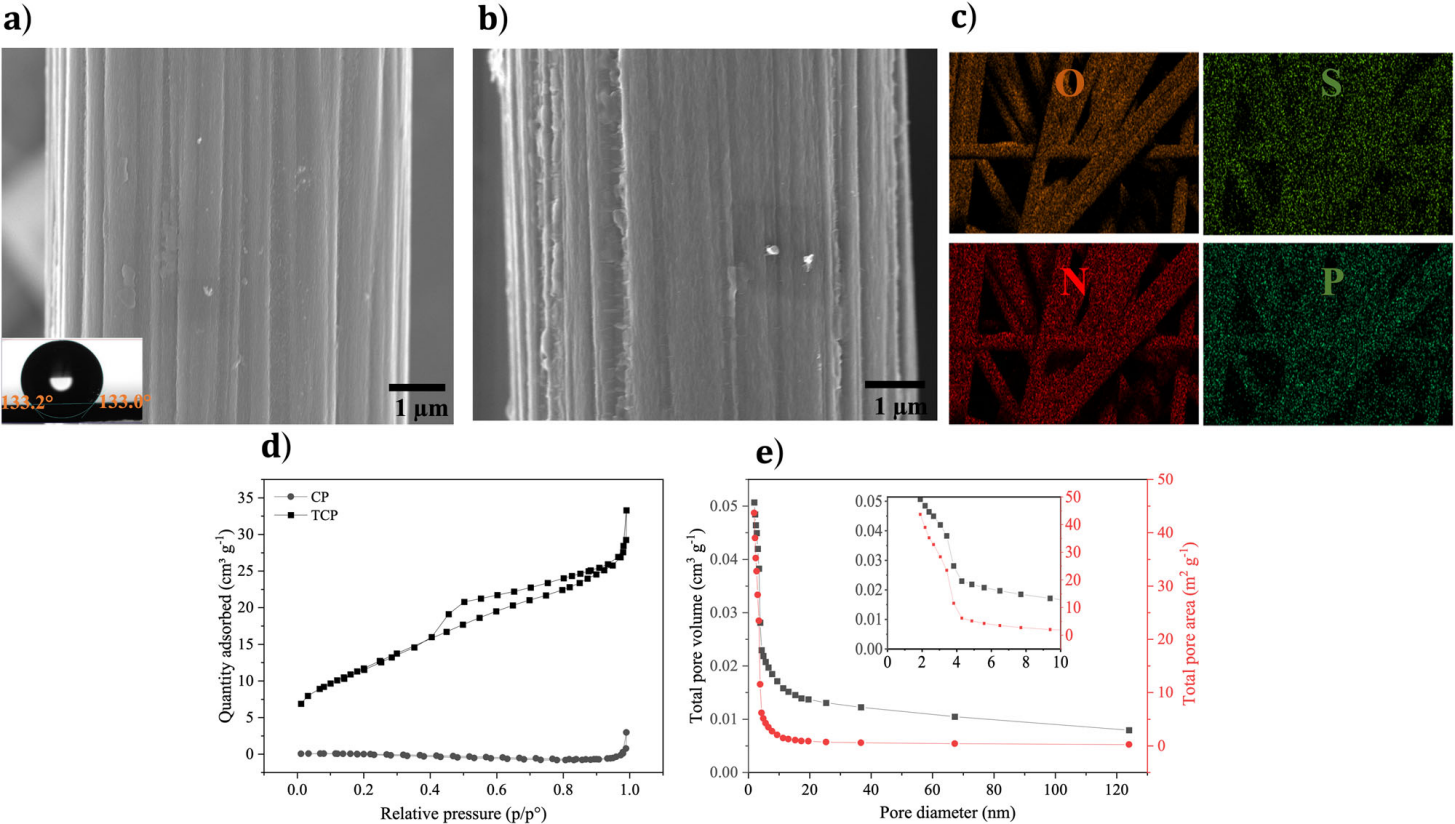
Morphology, elemental composition, surface area and pore size distribution of CP and TCP. SEM images: top view of (**a**) CP, (**b**) TCP, (**c**) EDX mapping of O, N, S, and P elements for TCP at 5 keV, (**d**) N_2_ adsorption/desorption isotherms for carbon paper (CP) and treated carbon paper (TCP), and (**e**) pore size distribution: total pore volume, total pore area, and pore diameter of TCP. Inset Fig. 1e: micropore and mesopore distributions of TCP.

To study the wettability of carbon with respect to aqueous electrolytes, we performed contact angle (wetting angle) measurements. CP exhibits a contact angle of about 133°, typical of a hydrophobic surface (inset of Fig. 2a and Supplementary Video 1). On the other hand, water droplets rapidly disappear on TCP, indicating that the treatment results in a hydrophilic surface (Supplementary Video 2).

To gain insight into surface area and pore size distribution, we performed N_2_ adsorption-desorption isotherm measurements using BET and BJH methods, respectively. TCP exhibits larger N_2_ adsorption compared to CP: the surface area increases from 0.4 m^2^ g^−1^ to 43.0 m^2^ g^−1^ and the pore volume increases from $6\times {10}^{-4}$ cm^3^ g^−1^ to $2.0\times {10}^{-3}$ cm^3^ g^−1^ (Supplementary Table 2). The adsorption isotherm of TCP shows a hysteresis loop, attributable to capillary condensation (Fig. 2d). In addition, the increase of N_2_ adsorption at high pressure suggests the co-existence of micro- (<2 nm), meso- (2–50 nm), and macro-pores (>50 nm)^57,58^. The pore size distribution analysis of TCP reveals a large majority of micropores (<2 nm) and mesopores (Fig. 2e). Pore diameters are primarily in the range of 1–4 nm, suitable for adsorption of hydrated SO_4_^2–^ (7.33 Å) and Na^+^ (3.59 Å) ions (inset Fig. 2e)^59^. The carbon architecture with porosities at different scales exhibits multiple advantages for energy storage: micropores provide active sites for ion adsorption and charge accumulation, mesopores provide a facile pathway for ion transport to minimize the capacitance fading at large current densities, and macropores serve as ion-buffering reservoirs that ensure ion availability for transport^57,58^.

EDX mapping shows the presence of O, N, S, and P on TCP, differently from CP, on which is shown only the presence of C with very small traces of O and N (Fig. 2c and Supplementary Fig. 1d). To further investigate the chemical effects of the treatment on carbon paper, XPS spectra were collected too (Supplementary Note 1, Supplementary Fig. 2 and Supplementary Table 3).

XRD spectra of CP and TCP show the characteristic graphitic peaks (002) located at 25.5° and (004) located at 54° (Supplementary Fig. 3a). CP shows a higher degree of graphitization than TCP through a more intense (002) peak and a decreased (002) interplanar distance (about 3.45 Å for CP and 3.49 Å for TCP)^60^. The lower graphitization degree of TCP could be attributed to the chemical surface treatment.

In addition, Raman spectra of CP and TCP show a graphitic-band and defect-band located at 1580 and 1350 cm^−1^, respectively (Supplementary Fig. 3b). A higher degree of disorder is observed for TCP with respect to CP, as TCP has a higher I_D_/I_G_ ratio (0.88 for TCP and 0.39 for CP), attributable to the chemical treatment.

#### Sepia and Tannins on Carbon

**Fig. 3:**
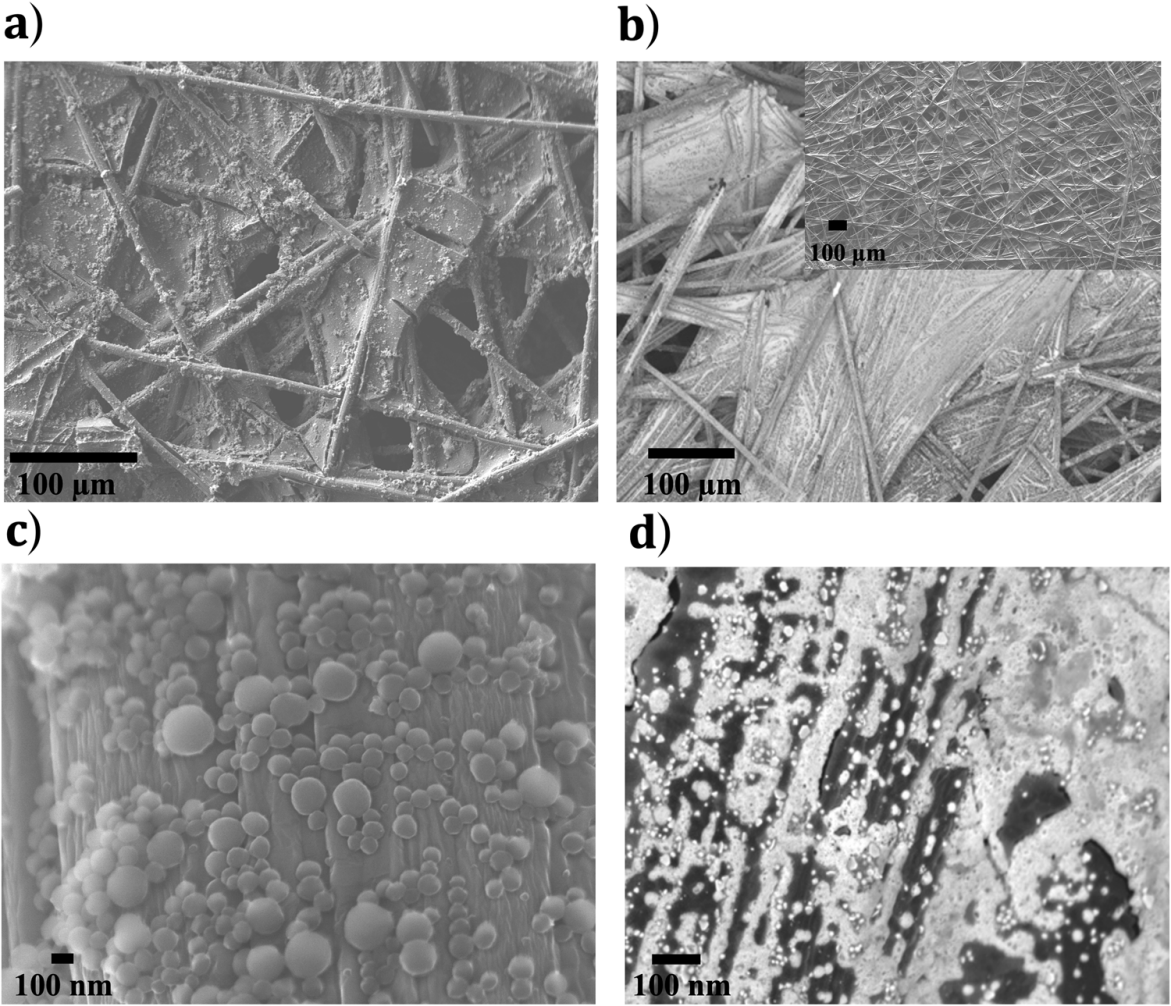
SEM images of quinone-based species on TCP. Top view of (**a**) sepia, (**b**) silver-stained catechin on TCP while (**c**) and (**d**) are tilted, zoomed-in views of samples in (**a**) and (**b**) at 5 keV. Inset (Fig. 4b) is the top-view image of unstained catechin on TCP.

SEM images obtained from sepia on TCP samples revealed dense spherical sepia aggregates (Fig. 3a, c)^61^. Since SEM images showed that catechin was not distinguishable from TCP (inset Fig. 3b), we stained the catechin with silver nitrate solution (Fig. 3b)^62^. High magnification images showed a heterogenous distribution of silver nanoparticles on TCP as a result of chemical reduction of silver cations by catechin molecules (Fig. 3d)^44,63^. It is worth noting that neither CP nor TCP show any bright regions attributable to the presence of silver after exposure to silver nitrate solution (Supplementary Fig. 1).

XPS survey spectra of sepia and Ctn on TCP clearly confirm the presence of biosourced quinones on TCP through the coexistence of carbon (C 1 s), oxygen (O 1 s), nitrogen (N 1 s), and sulfur (S 2 s and S 2p) (Supplementary Note 2, Supplementary Figs. 4 and 5).

#### Electrochemical Characterization in 3-Electrode Cell Configuration

**Fig. 4:**
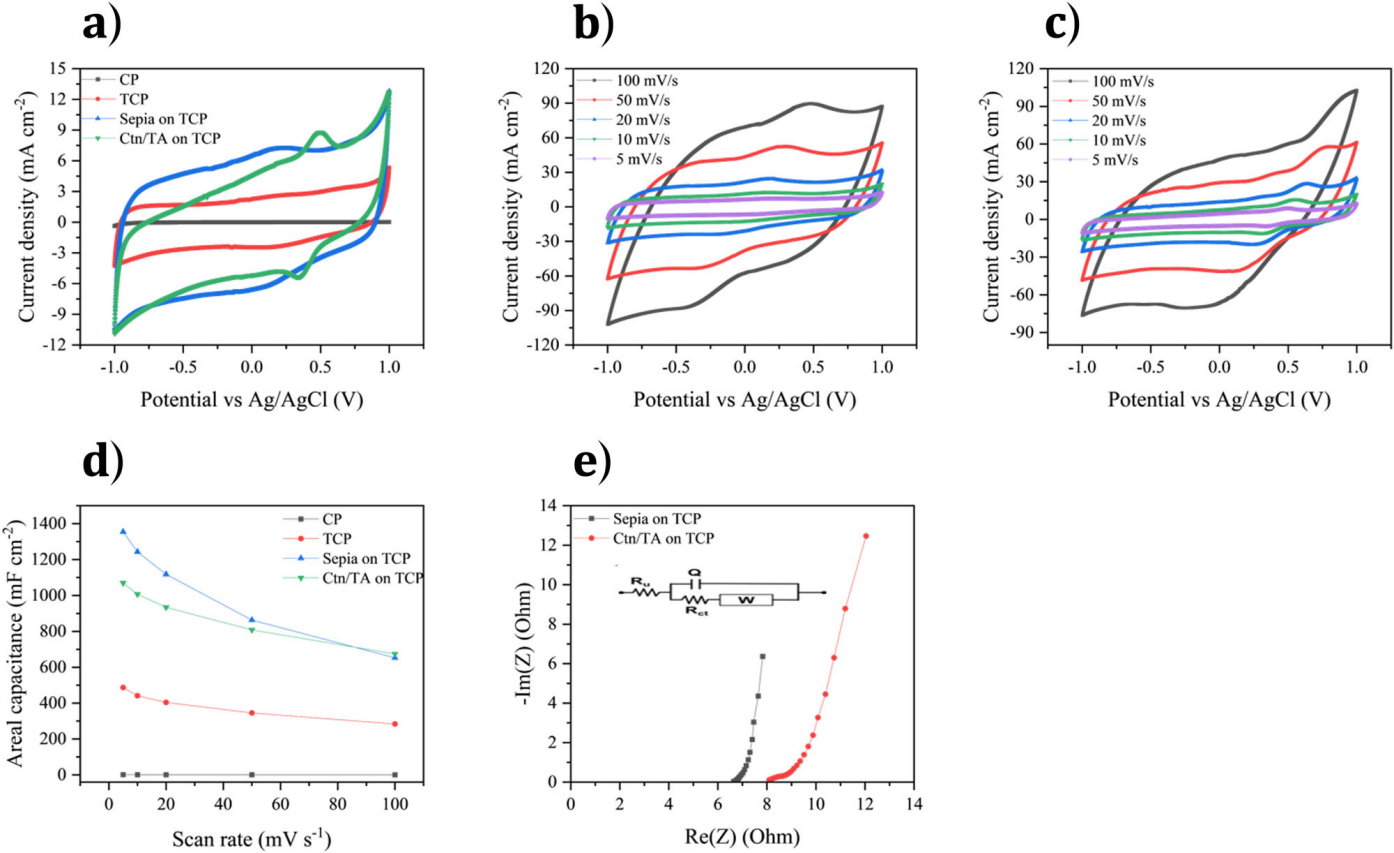
Electrochemical characterization of bare CP, bare TCP, and sepia on TCP and Ctn/TA on TCP. (**a**) Cyclic voltammetry at 5 mV s^−1^. Cyclic voltammetry at different scan rates of (**b**) sepia on TCP, (**c**) Ctn/TA on TCP. (**d**) Capacitance vs scan rate for the aforementioned samples, obtained from cyclic voltammetry. (**e**) Nyquist plot for sepia and Ctn/TA on TCP in the frequency range 4.25 × 10^3^−10^−1^ Hz. Inset: corresponding simplified simulated circuit where R_u_ is the uncompensated resistance, R_ct_ is the charge-transfer resistance, Q is the constant phase element, and W is the Warburg element.

The electrochemical behavior of CP, TCP, and sepia or Ctn on CP and TCP was studied through cyclic voltammetry and electrochemical impedance spectroscopy in 0.5 M Na_2_SO_4_ aqueous electrolyte. The CVs show a quasi-box-shaped CV and an electrochemical stability window of ca. 2 V (no obvious oxygen or hydrogen evolution is observable at the electrodes, Fig. 4a–c). The wide electrochemical window in a mild-pH aqueous electrolyte can be tentatively ascribed to several factors, such as low [H^+^] and [OH^−^], water molecules engaged in a strong solvation of ions such as Na^+^, and carbon surface partly covered by adsorbed ions of the electrolyte. This contributes to the increased overpotential for decomposition of the water molecules at the surface of the electrode^59,64^.

TCP shows better stability in the cathodic potential region and two orders of magnitude higher voltammetric current compared to CP (Fig. 4a, Supplementary Fig. 6a, b). At 5 mV s^−1^, TCP shows an areal capacitance of ca. 500 mF cm^−2^ compared to 1.2 mF cm^−2^ for CP (Fig. 4d). Several factors could explain why the treatment improves the electrochemical performance. Besides increased surface area and suitable porosity, the heteroatoms (O, N, S, and P) bring in a polar electrode surface with enhanced electrolyte wettability (i.e. ion adsorption) and possible Faradic (charge transfer) processes at the TCP surface^65,66^.

Electrochemical impedance spectroscopy (EIS) not only confirms the capacitance enhancement for TCP with respect to CP but also sheds light onto the aforementioned Faradic processes. EIS plots were fitted according to the Randles equivalent circuit (inset of Fig. 4e). TCP exhibits a small semicircle as a result of Faradic processes attributable to the presence of the heteroatoms; this semicircle is absent for CP (Supplementary Fig. 6c). The charge transfer resistance, quantified from the diameter of the semicircle, is 0.3 ohms. Further, the high-frequency intercepts of the Nyquist plots of CP and TCP are different (Supplementary Fig. 6c); such intercepts are affected by the electronic resistance of the working electrode, bulk electrolyte resistance and cell geometry (distance between the reference and working electrode). TCP features a more vertical low-frequency diffusion line with a lower imaginary impedance component than that of CP (8 Ohms vs. 16.5 kOhms) (Supplementary Fig. 6c). This reflects the better capacitive behavior of TCP compared to CP.

After the electrochemical characterization of CP and TCP electrodes, we proceeded to the characterization of electrodes made of quinone-based biosourced materials on CP and TCP.

CV curves of sepia on TCP, obtained at 5 mV s^−1^, show about one order of magnitude higher voltammetric current than that for sepia on CP and redox features located at about 0.16 and 0.09 V vs. Ag/AgCl (Fig. 4a and Supplementary Fig. 7b). Those features are attributable to the hydroquinone-quinone redox couple (Fig. 1a)^67^. Sepia on CP features broad redox features at about 0.11 and 0.02 V vs. Ag/AgCl (inset of Supplementary Fig. 7b).

Catechin on TCP shows redox features at ca. 0.50 and 0.45 V vs. Ag/AgCl (Fig. 4a and Supplementary Fig. 8b), also attributable to the hydroquinone-quinone redox couple (Fig. 1b)^68^. These features are consistent with those observed in other quinone-based plant varieties such as bark tannins^27^. Catechin redox peaks are also observable on CP (Supplementary Fig. 8c). The limited cycling stability of catechin, due to its high solubility in aqueous electrolytes, prompted us to mix catechin with tannic acid, a tannin-based binder with high hydrogen-bonding capability^54^. After mixing, the redox activity of tannic acid does not significantly affect the redox activity of catechin (Supplementary Fig. 8g, obtained at 5 mV s^−1^).

The CV curves of sepia and Ctn/TA on TCP at different scan rates (5 to 100 mV s^−1^) confirm the capacitive behavior of the electrodes (Fig. 4b, c) and suggest efficient electronic coupling between biosourced quinones and TCP^30,67^. EIS of sepia and Ctn/TA on TCP confirms enhanced capacitance and decreased charge transfer resistance compared to their deposition on CP (Fig. 4e, Supplementary Figs. 7c and 8h).

The deposition of the biosourced materials on TCP had a significant effect on the capacitance and the charge-transfer resistance^22^. The capacitance of sepia on TCP increased from 38 to 1355 mF cm^−2^ (13 to 452 F g^−1^, based on the mass of quinone-based material) with respect to CP, while the charge-transfer resistance decreased from 4 to 0.15 ohms cm^−2^ (Supplementary Fig. 7h, i).

For Ctn/TA, the capacitance on TCP increased from 21 to 898 mF cm^−2^ (7 to 300 F g^−1^, based on the mass of quinone-based material) compared to CP, whereas charge-transfer resistance decreased (from 10.8 to 1.4 ohms cm^−2^) (Supplementary Fig. 8k, l).

Both sepia and Ctn/TA on TCP maintain good rate capabilities, achieving 670 mF cm^−2^ (223 F g^−1^) and 680 mF cm^−2^ (227 F g^−1^), respectively, at 100 mV s^−1^, compared to 1355 mF cm^−2^ (452 F g^−1^) and 898 mF cm^−2^ (300 F g^−1^) at 5 mV s^−1^, respectively (Fig. 4d). This performance can be attributed to facile charge transfer, in turn due to effective electronic coupling between TCP and sepia or Ctn/TA, facile ion transport and availability of an ion buffering reservoir within the porous architecture of TCP. These results confirm the success of interface engineering by treating carbon prior to deposition of the quinones to boost energy storage.

Our biosourced quinones on CP show a Faradic (pseudocapacitance) contribution of about 95% to the total capacitance with respect to non-Faradic (electric double layer). Upon deposition on TCP, sepia and Ctn/TA show a non-Faradic contribution (electric double layer) of about 73% and 85%, respectively, to the total capacitance with respect to Faradic (pseudocapacitance)^69^. This confirms the hybrid nature of our electrode materials and explains their interesting electrochemical properties (Supplementary Note 3, Evaluation of the Faradic and non-Faradic capacitance contribution, Supplementary Fig. 9).

It is worth noting that mixing biosourced quinones with common conductive additives (e.g., conductive carbon super P (SP) and reduced graphene oxide (r-GO)) did not bring any significant performance storage improvement (Supplementary Note 4, Supplementary Figs. 10, 11, and 12), differently from depositing the biosourced quinones without additives on TCP.

#### Characterization of symmetric devices based on treated carbon and biosourced quinones on treated carbon paper (TCP)

After performing voltammetric and impedance studies on electrode materials, we assembled symmetric supercapacitors and proceeded to their characterization to examine the suitability of our electrode materials for energy storage applications.

**Fig. 5:**
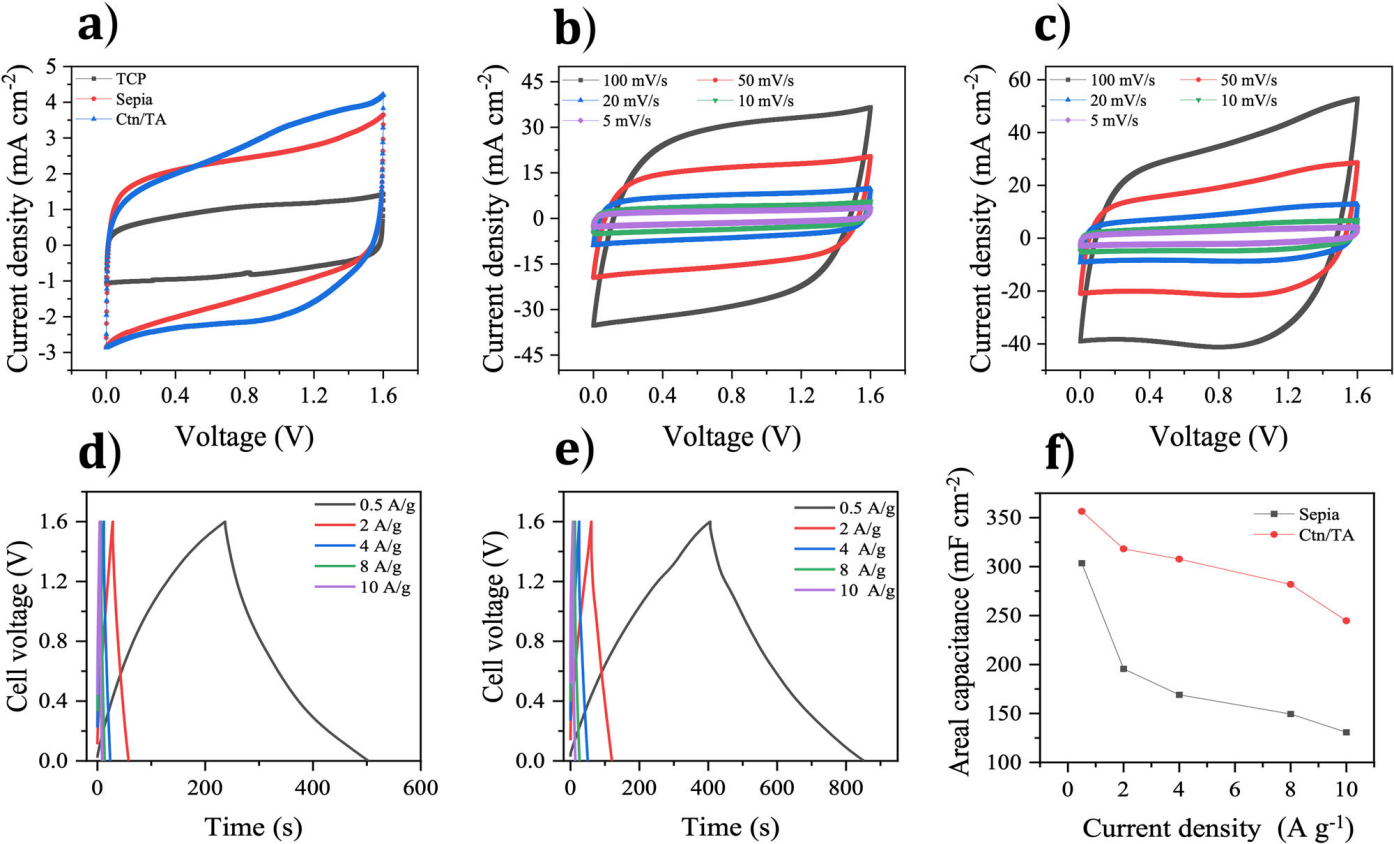
Electrochemical characterization of symmetric supercapacitors based on TCP, sepia on TCP, and Ctn/TA on TCP in 0.5 M Na_2_SO_4_. (**a**) Cyclic voltammetry at 5 mV s^−1^. (**b**) Cyclic voltammetry of sepia on TCP at different scan rates. (**c**) Cyclic voltammetry of Ctn/TA on TCP at different scan rates. Galvanostatic charge/discharge curves at different current densities of (**d**) sepia on TCP and (**e**) Ctn/TA on TCP. (**f**) Relationship between areal capacitance evaluated from galvanostatic charge/discharge and corresponding current density.

The CV curves of TCP supercapacitors clearly show lower voltammetric currents and lower coulombic efficiency (ca. 99%) compared to sepia and Ctn/TA on TCP (Fig. 5a and Supplementary Fig. 13d). The voltammograms of sepia and Ctn/TA on TCP devices at different scan rates are typical of pseudocapacitive supercapacitors (Fig. 5b, c).

The supercapacitors should be able to withstand a voltage window of 2 V, based on the CV curve in the three-electrode cell configuration. However, for long-term stability of the device, we shrank the operating voltage to 1.6 V to limit possible cycling-induced overoxidation/overreduction of the active material and to ensure reversible charging/discharging processes at high current density (Supplementary Fig. 13a–c). Galvanostatic charge/discharge curves of sepia and Ctn/TA supercapacitors at high current density (150 mA cm^−2^ i.e. ~10 A g^−1^) feature a nearly triangular shape, indicating reversible pseudocapacitive behavior with excellent coulombic efficiency (ca. 100%) (Fig. 5d–e and Supplementary Fig. 14).

The specific capacitance decreases with increasing current density for both sepia and Ctn/TA supercapacitors. This is attributable to ion diffusion-limited transport at higher current densities (Fig. 5f). Nevertheless, we observed that both sepia and Ctn/TA supercapacitors work at high specific currents (30 to 150 mA cm^−2^ ~2 to 10 A g^−1^), with a decrease in areal capacitance of less than 25%. The areal capacitance of the symmetric supercapacitors based on *Sepia* on TCP decreases with current density more rapidly than that one of those based on Ctn/TA on TCP (Fig. 5f). The spherical *Sepia* nano-aggregates could limit the access of ions to the carbon’s pores. Such a limitation is not expected to be present in the case of the small molecules Ctn and TA deposited on carbon’s surface.

**Fig. 6:**
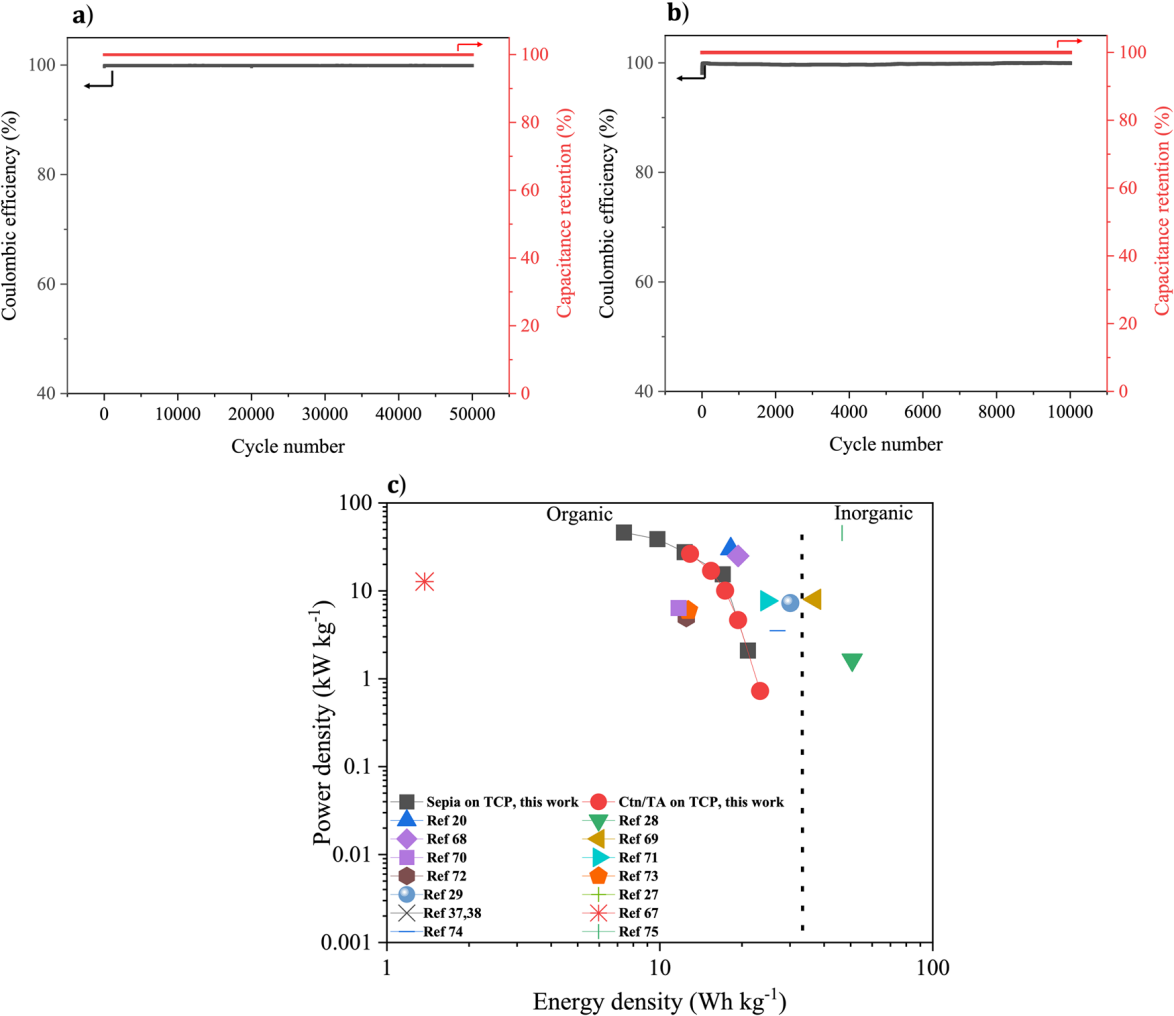
Electrochemical characterization of sepia and Ctn/TA symmetric supercapacitors deposited on treated carbon in 0.5 M Na_2_SO_4_. Capacitance retention and coulombic efficiency for 50 000 and 10 000 cycles of galvanostatic charge/discharge at 10 A g^−1^ of (**a**) *Sepia* on TCP and (**b**) Ctn/TA on TCP. (**c**) is the Ragone plot extracted from galvanostatic charge/discharge cycles at different current densities: 0.5, 2, 4, 8, and 10 A g^−1^, compared with the state of the art in literature. See refs. ^22,29–31,39,40,69,71–78^ and table S1 for a more comprehensive study that includes a wider range of organic, inorganic, and hybrid materials.

*Sepia* and Ctn/TA supercapacitors feature remarkable cycling stabilities (ca. 100% capacitance retention) and coulombic efficiencies (ca. 100%) over 50,000 cycles for *Sepia* and 10,000 cycles for Ctn/TA at 10 A g^−1^ (Fig. 6a, b).

Galvanostatic charge/discharge cycles of sepia and Ctn/TA supercapacitors feature a small potential drop at the beginning of the discharge ($\triangle V$, ca. 21 and 29 mV, respectively, at 0.5 A g^−1^), corresponding to a low equivalent series resistance (ca. 1 and 2 ohm cm^−2^, Fig. 5d, e and Supplementary Fig. 15). Ragone plots of sepia and Ctn/TA supercapacitors illustrate the practical energy densities and power densities of the devices at different values of current density (Fig. 6c). These devices exhibit noteworthy maximum energy densities of ca. 0.56 and 0.65 mW h cm^−2^ ~20 and 23 W h kg^−1^ and maximum power densities of ca. 1274 and 727 mW cm^−2^ ~46 and 26 kW kg^−1^, respectively. Specific values refer to the total mass (current collector and quinone-based material, for the whole device). These figures of merit are rarely obtained with organic biosourced materials without a conductive additive or fluorinated binder (please refer to the literature review reported in Fig. 6c and Supplementary Table 1). They are attributed to the increased surface area, improved wettability, and enhanced conductivity of sepia and Ctn/TA on TCP electrode material that make the surface area of the electrode easily accessible to electrolyte ions.

## Conclusions

Biodegradable, biosourced redox-active organic quinone-based materials deposited on carbon, operating in mild-pH aqueous electrolytes, represent a promising option for resilient green energy storage.

Unfortunately, issues such as high contact resistance at the organic material/carbon interface often lead to poor rate response and short cycling stability. We show that engineering such an interface, e.g., by chemically treating carbon paper prior to deposition of the quinone-based material, improves rate response and cycling stability by bringing about increased surface area, suitable porosity, and improved aqueous electrolyte wettability of the carbon surface. Further, the O, N, S, and P surface-doping of carbon, after the chemical treatment, imparts Faradic activity to the carbon surface that parallels the typical electrostatic activity of carbon. The deposition of two biosourced quinone-based materials, *Sepia* melanin and catechin/tannic acid, on treated carbon paper (TCP) results in the improvement of capacitance by a factor of two to three with respect to bare TCP without requiring any conductive additive or toxic binder. Tannic acid was introduced as a binder with hydrogen-bonding capability in order to limit the solubility of catechin in aqueous electrolytes.

Symmetric electrochemical capacitors were fabricated with *Sepia* melanin (extracted from cuttlefish ink) and catechin/tannin acid. *Sepia* melanin reached capacitance of 1355 mF cm^−2^ (452 F g^−1^), 100% coulombic efficiency, and ca. 100% capacitance retention after 50 000 cycles. Ctn/TA reached capacitance of 898 mF cm^−2^ (300 F g^−1^), 100% coulombic efficiency, and about 100% capacitance retention after 10 000 cycles. These devices exhibited noteworthy maximum energy and power densities: 0.56 and 0.65 mW h cm^−2^ (20 and 23 W h kg^−1^, based on the total mass of the current collector and quinone-based material for the whole device) and 1274 and 727 mW cm^−2^ (46 and 26 kW kg^−1^) for sepia and catechin/tannic acid, respectively.

Our work paves the way to high-performance sustainable electrochemical capacitor electrodes based on biosourced and biodegradable organic materials. We are currently investigating other organic materials extracted from natural sources to demonstrate the universality of our interface engineering approach. Mechanically robust, environmentally friendly, and biodegradable supercapacitors making use of hydrogels, instead of liquid electrolytes, are currently under investigation.

## Methods

### Treatment of carbon paper

Carbon paper (CP) was purchased from Fuel Cell Store (Spectracarb 2050A-1550, 10 mils, plane electrical resistivity of 5.4 mΩ cm, composed of multiple plies of graphitized resin-bonded carbon fibers). Carbon paper was cut in 5-cm-by-0.5-cm rectangular pieces, cleaned sequentially in anhydrous ethanol (Commercial Alcohols, Ontario, Canada) and acetone (Honeywell, VLSI, 100%) in an ultrasonic bath at 40 kHz (Eumax-4L), and dried under vacuum for 30 min. at 60˚C. The cleaned carbon was activated through a two-step oxidative treatment. First, it was sonicated at 40 kHz for 2 h in a mixed acid solution (30 mL H_2_SO_4_ (Sigma-Aldrich ACS reagents 95%–98%): 10 mL HNO_3_ (16 M Fischer Chemical, ACS plus)) and placed, in the same solution, in an autoclave for a thermal treatment of 20 min. at 120˚C followed by cooling to room temperature. Second, the previously treated carbon was rinsed with deionized water (MilliQ water, 18.2 MΩ m) and placed in an autoclave for a thermal treatment of 24 h at 180˚C in a 7 M (NH_4_)_2_HPO_4_ (Sigma-Aldrich, ACS reagents >98%) saturated solution. After cooling to room temperature in the autoclave, the treated carbon was rinsed with deionized water and dried under vacuum for 6 h at 60˚C to produce what we indicate as treated carbon paper (TCP).

### Electrode preparation

*Sepia* melanin was extracted from the ink sac of the cuttlefish *Sepia officinalis* (commercially available in the fish market) then purified and ground into a fine powder^70^. Catechin (Ctn) hydrate and tannic acid (TA) were purchased from Sigma-Aldrich (ACS reagents >98%). Reduced graphene oxide (r-GO from Sigma-Aldrich) and Super P carbon black (SP, Imerys Graphite & Carbon) were used as conductive additives in the preparation of some types of electrodes.

Sepia, sepia/r-GO, sepia/SP, and Ctn/r-GO electrodes were prepared by mixing the active molecule (sepia or catechin), the chosen conductive additive (r-GO or SP) in different mass ratios of 8:2,7:3, 6:4, and 5:5, and a few drops (70 µL for 25 mg of composite powder) of dimethyl sulfoxide (DMSO) (Sigma-Aldrich anhydrous, ≥ 99.9%). The preparation was stirred overnight to create a uniform slurry that was deposited over TCP (covering 1-cm-by-0.5-cm of the 5-cm-by-0.5-cm rectangular pieces) with a brush.

Ctn/TA/SP and Ctn/TA electrodes were prepared by mixing the materials in powder form with mass ratios of 7:1:2 and 7:1, respectively, in a deionized water-ethanol mixture (2:1 v/v) (1 mL of solvent for 50 mg of composite powder) and stirring vigorously to form a homogenous solution. Afterwards, 63 µL of the solution were drop-cast over TCP (same coverage as the other electrodes).

All electrodes were vacuum-dried for 20 min at 60˚C prior to morphological and electrochemical characterizations. The loading of active material in all electrodes was about 3.0 ± 0.2 mg cm^−2^ on TCP (the mass of bare TCP for the covered surface was 4.74 mg ± 0.20 mg cm^−2^), measured using a microbalance (Sartorius BP 210 D, accuracy 10^–5^ g).

### Electrolyte

0.5 M Na_2_SO_4_ aqueous solutions (pH ca. 5) were prepared from Na_2_SO_4_ (Sigma-Aldrich >99%) dissolved in DI water (18.2 MΩ cm).

### Electrochemical characterization

#### For material characterization

cyclic voltammetry (CV), galvanostatic charge/discharge (GCD), and electrochemical impedance spectroscopy (EIS) measurements were performed using a Biologic bipotentiostat (SP-300) in a three-electrode cell configuration, with CP or TCP loaded with active materials as working electrodes, Pt mesh as a counter electrode, and Ag/AgCl in 3 M NaCl as a reference electrode.

CV was performed in the potential range of −1 V to 1 V vs. Ag/AgCl at scan rates of 100, 50, 20, 10, and 5 mV s^−1^. EIS measurements were conducted before and after the CV scans in the same setup at open circuit potential and 10 mV AC amplitude within the frequency range 10^5 ^Hz to 10^−1 ^Hz.

#### Symmetric supercapacitor characterization

CV, GCD, and EIS were performed with TCP loaded with active material as working and counter electrodes. These two electrodes were separated by a filter paper. Ag/AgCl reference electrode was used to monitor the potential of each electrode during the tests.

GCD was performed at current densities of 0.5, 2, 4, 8, and 10 A g^−1^ (calculated over the total mass of the current collector and quinone-based material for the whole device) for a potential scan ranging from 0 V to 1.6 V vs. Ag/AgCl. Finally, 50 000 and 10 000 GCD cycles were performed at a current density of 10 A g^−1^ for sepia and Ctn/TA supercapacitors, respectively.

The electrode-specific capacitance was evaluated from 3-electrode CV measurements using:

$${{{{{{\rm{C}}}}}}}_{{{{{{\rm{CV}}}}}}}=\frac{\int {{{{{\rm{I}}}}}}\,{{{{{\rm{dV}}}}}}}{{{{{{\rm{\nu }}}}}}\,{{{{{\rm{w}}}}}}\,\triangle {{{{{\rm{V}}}}}}}$$

Where $\int I{{{{{\rm{d}}}}}}V$ is the integral area of the cathodic (discharge) CV cycle, ${{{{{\rm{\nu }}}}}}$ the scan rate, $w$ the mass loading of the active material on the current collector, and $\triangle V$ the potential range.

From GCD curves, the cell-specific capacitance (${C}_{{{{{{\rm{GCD}}}}}}}$), coulombic efficiency (${{{{{\rm{\eta }}}}}}$), equivalent series resistance (${{{{{\rm{ESR}}}}}}$), power density ($P$), energy density ($E$), maximum power density (${P}_{{{\max }}}$), and maximum energy density (${E}_{{\max }}$) were calculated at different current densities using:

$${{{{{{\rm{C}}}}}}}_{{{{{{\rm{GCD}}}}}}}	=\frac{{I}_{{dis}}\int \left(\frac{1}{V}\right){dt}}{{{{{{\rm{w}}}}}}},\,{{{{{\rm{\eta }}}}}}=\frac{\int {I}_{{dis}}\,{dt}}{\int {I}_{{ch}}{dt}},{{{{{\rm{ESR}}}}}}=\frac{{\triangle {{{{{\rm{V}}}}}}}_{{ESR}}}{2\,{I}_{{dis}}},E=\frac{{I}_{{dis}\int {Vdt}}}{3600},\\ {{{{{\rm{P}}}}}}	=\frac{E}{{{{{{{\rm{t}}}}}}}_{{dis}}},{{{{{{\rm{E}}}}}}}_{{{\max }}}=\frac{1/2{{{{{{\rm{C}}}}}}}_{{{{{{\rm{GCD}}}}}}}{{{{{{\rm{V}}}}}}}_{{{\max }}}^{2}}{3600}\ {{{{{\rm{and}}}}}}\ {{{{{{\rm{P}}}}}}}_{{{\max }}}=\frac{{{{{{{\rm{V}}}}}}}_{{{\max }}}^{2}}{4\,{{{{{\rm{ESR}}}}}}\,2{{{{{\rm{w}}}}}}}$$

Where, ${I}_{{dis}}$ and ${I}_{{ch}}$ are the constant discharge and charge currents, respectively, ${t}_{{dis}}$ is the discharging time, ${\triangle V}_{{ESR}}$ the ohmic drop at the beginning of the discharge, $\int {Vdt}$ the integral area of the GCD discharge cycle, and ${V}_{{\max }}$ the upper limit of the potential while charging (charge cut-off potential).

At least five samples for each electrode material and 3 symmetric supercapacitor devices of TCP, sepia on TCP, and Ctn/TA on TCP were tested. All of them (electrode materials and supercapacitors) give the same electrochemical response with almost the same performance, with an error of ± 5%.

### Morphology and structure characterization

The morphology of the sepia, CP, and TCP was examined by scanning electron microscopy (SEM, JEOL JSM-7600F) at an acceleration voltage of 5 kV. The morphology of Ag-stained Ctn/TA composite was examined in both secondary and backscattered electron modes at 5 kV. The Ctn/TA electrodes were stained in 0.5 M AgNO_3_ for 48 h prior to morphology examination by SEM^44,63^. Energy-dispersive X-ray spectroscopy (EDX) was carried out using the same SEM with Aztec (Oxford) software and detector x-Max (80 mm^2^) (Oxford), at 5 kV. X-ray diffraction was carried out on a Bruker D2-Phaser X-ray diffractometer using Cu Kα radiation generated at 30 kV.

The elemental composition of the samples was studied by X-ray photoelectron spectroscopy (XPS), using a VG ESCALAB 2250 apparatus. The X-ray source was Al Kα (1486.6 eV) at a power of 1 W (1 kV, 1 mA). Pressure in the analysis chamber was lower than 10^-9^ mbar. Survey scans and high-resolution scans were carried out with 1.0 eV and 0.1 eV energy steps, respectively.

Raman spectra of CP and TCP were acquired using Raman microscope Senterra (Bruker), furnished with laser excitation at 532 nm. Spectra were recorded in optical geometry 180° in the range of Raman shifts 100–3200 cm^−1^ at optical resolution of 3–5 cm^−1^, using a laser excitation power of 20 mW.

### Brunauer-Emmett-Teller surface area, pore volume, and pore size measurements

Brunauer-Emmett-Teller (BET) surface area, pore volume, and pore size of CP and TCP were evaluated by N_2_ adsorption/desorption measurement (Micromeritics, model TriStar 3000). Samples were first degassed at 120 °C under vacuum overnight, and then analysis was carried out using N_2_ as an adsorbate gas at −196 °C; the volume of the adsorbate gas was determined at standard temperature and pressure (STP) (273.15 K and atmospheric pressure (1.013 × 10^5 ^Pa)). Surface area and pore-size distribution were determined by BET and Barrett-Joyner-Halenda (BJH) methods, respectively.

### Contact angle measurements

Contact angle measurements for CP and TCP, according to sessile and captive drop methods, were performed using DataPhysics dynamic contact-angle-measuring devices and a force tensiometer. 2 µL water droplets were used at a speed rate of 2 µL s^−1^.

## Supplementary information

Supplementary Information

Description of Additional Supplementary Files

Supplementary Video 1

Supplementary Video 2

## Data Availability

All the data of this study are available. The authors declare that the data supporting the findings of this study are available within the article and its Supplementary Information files. The data that support the findings of this study are available from the corresponding authors upon reasonable request.
