# Supplementary material for: Biosourced quinones for high-performance environmentally benign electrochemical capacitors via interface engineering
Source: Commun Chem. 2022 Aug 20;5:98. doi: 10.1038/s42004-022-00719-y (PMC9814668; doi:10.1038/s42004-022-00719-y)
Supplement: Supplementary file 1 — Supplementary Information [file 42004_2022_719_MOESM1_ESM.docx]

**Supplementary Information**

**Biosourced Quinones for High-Performance Environmentally Benign Electrochemical Capacitors via Interface Engineering**

Abdelaziz Gouda^a,b*^, Alexandre Masson^a^, Molood Hoseinizadeh^a^, Francesca Soavi^c^ and Clara Santato^a*^

^a^Department of Engineering Physics, Polytechnique Montreal, C.P. 6079, Succ. Centre-ville, Montreal, Quebec, H3C 3A7, Canada

^b^ Now at, Solar Fuels Research Group, Department of Chemistry, University of Toronto, 80 St. George Street, Toronto, Canada, M5S 3H6

^b^Department of Chemistry “Giacomo Ciamician”, Alma Mater Studiorum Università di Bologna, Via Selmi, 2, Bologna, Italy 40126

Corresponding authors: Dr Abdelaziz Gouda and Prof. Clara Santato

[abdelaziz.gouda@utoronto.ca](mailto:abdelaziz.gouda@utoronto.ca) and [clara.santato@polymtl.ca](mailto:Clara.santato@polymtl.ca)


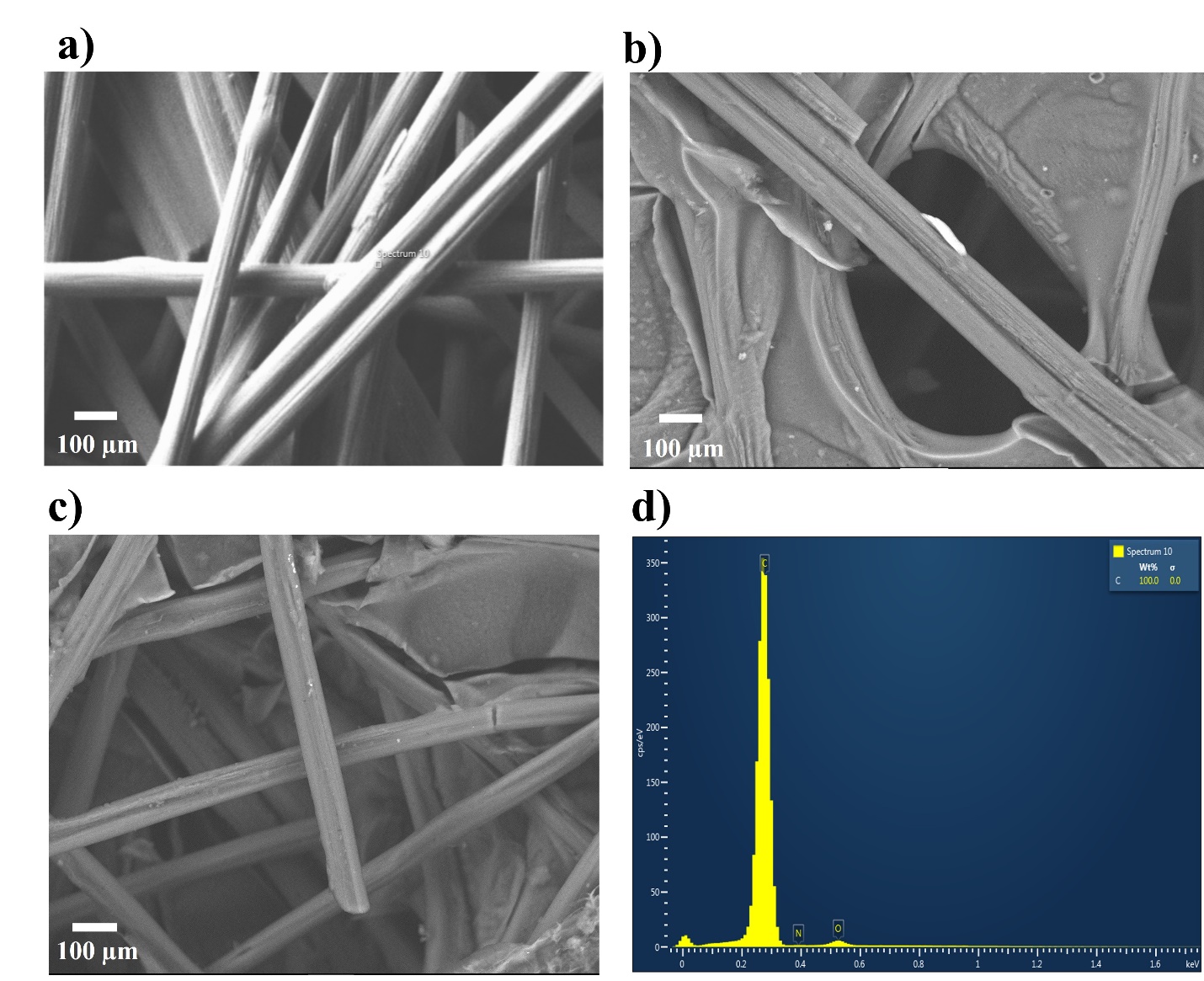


Supplementary Figure 1. Morphology and elemental analysis of carbon paper and silver-stained carbon paper. SEM images in top-view mode of (a) carbon paper, (b) silver-stained carbon paper, and (c) treated carbon paper. (d) EDX mapping of carbon paper.

**Supplementary Note 1: Surface chemical composition of carbon paper and treated carbon paper**

For TCP, the survey XPS spectra (Supplementary Fig. 2d) show peaks from P 2p, S 2p, O 1s (high intensity), N 1s, and C 1s. The C 1s peak is composed of C=C at binding energy of 284.3 eV, C−C at 284.8 eV, C−O at 286.1 eV, C=O at 287.8 eV, and O–C=O at 289.0 eV (Supplementary Fig. 2b). C−O and O−C=O are less present on CP (Supplementary Table 3), which is mainly composed of C=C and C−C bonds. C−O and C−C bonds are clearly present in TCP^1,2^. In addition, the O 1s spectrum shows signals from -OH bonding at 531.8 eV, COOH at 533.3 eV, and adsorbed water at 534.6 eV (Supplementary Fig. 2c). A more intense COOH peak is observed for TCP compared to CP. The N 1s peak consists of N−(C−O)−N at 399.9 eV, N−C at 401.9 eV, and NO_2_ at 406.4 eV (Supplementary Fig. 2e). We observe that the relative amount of N−C is greater for TCP compared to CP in N 1s spectra. The NO_2_ signal is only observed in the spectra of TCP. The S 2p spectrum indicates the presence of sulfone groups (C−SO_x_−C) at 167.20 and 169.0 eV (Supplementary Fig. 2f)^3^. Finally, the P 2p spectrum reveals two peaks at 131.6 and 133.3 eV corresponding to P−C and P−N bonds (Supplementary Fig. 2g)^4^. The size of the P atoms allows for the formation of longer P−C bonds (1.77Å) compared to C−C bonds (1.54 Å): this increases the surface area by distorting the carbon network, in turn improving carbon capacitance^5,6^.


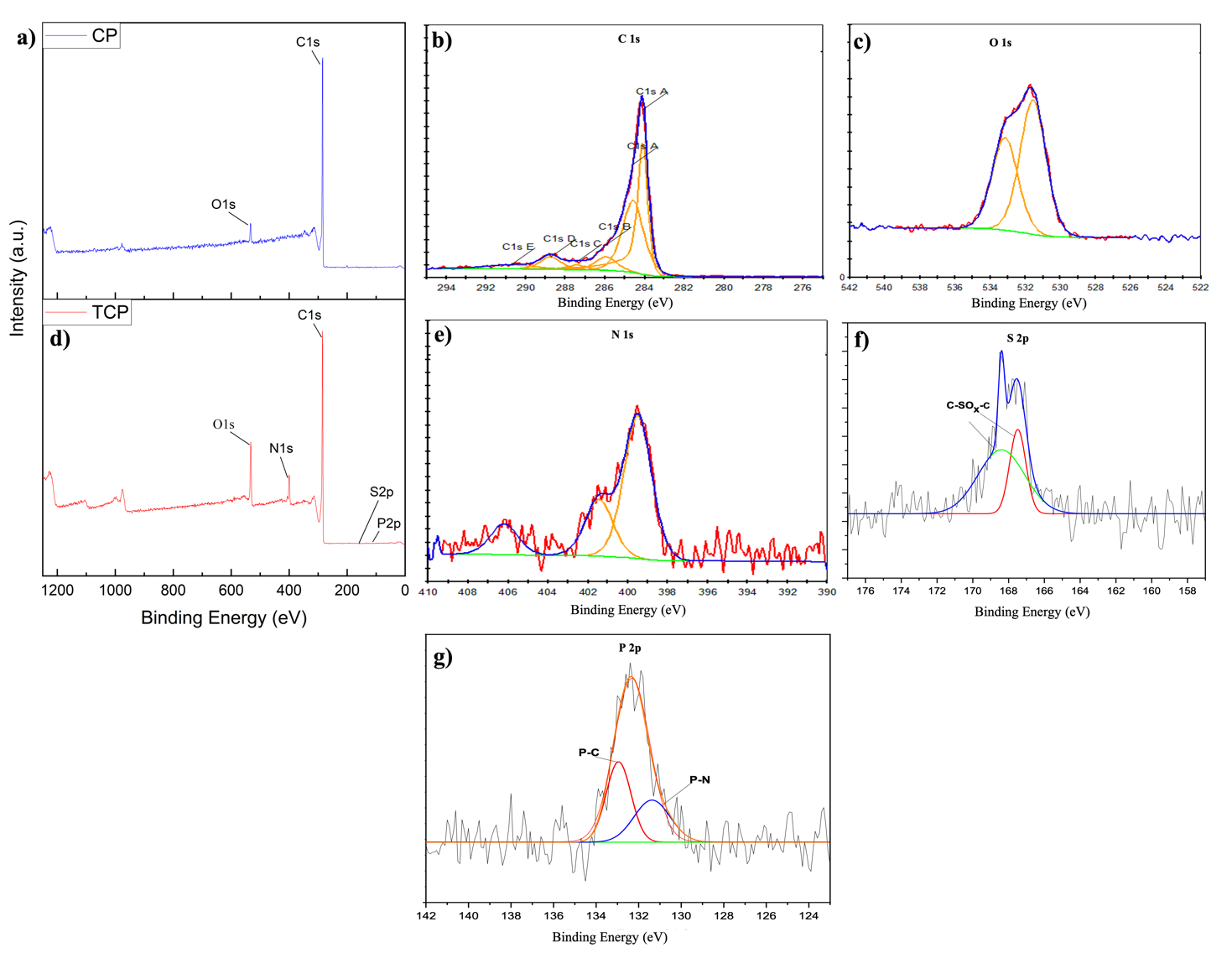


Supplementary Figure 2. Surface chemical composition of carbon paper and treated carbon paper. XPS survey spectra of (a) carbon paper (CP) and (d) treated carbon paper (TCP). Deconvoluted spectra: (b) C 1s, (c) O 1s, (e) N 1p, (f) S 2p, and (g) P 2p of treated carbon paper.

*
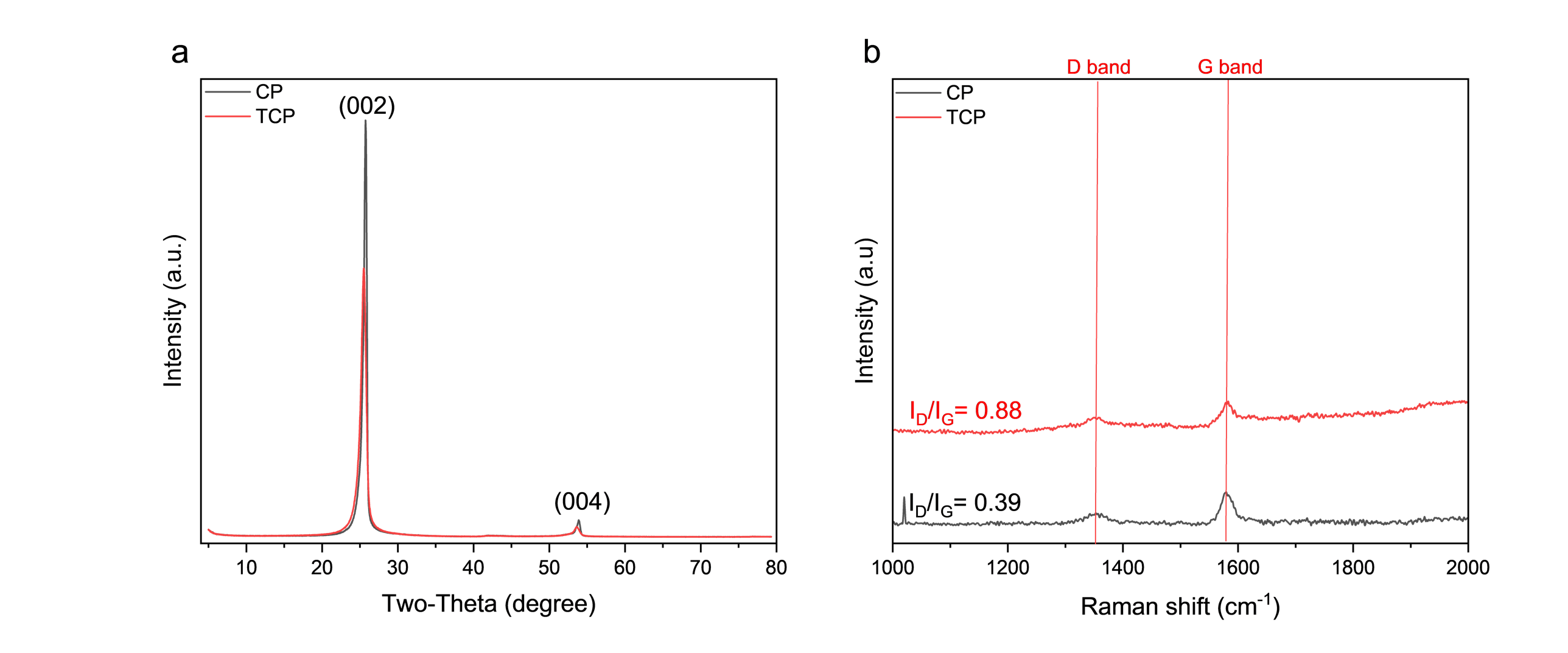
*

Supplementary Figure 3. Characterization of carbon paper (CP) and treated carbon paper (TCP). (a) XRD patterns and (b) Raman spectra, for CP and TCP.

**Supplementary Note 2: Surface chemical composition of Sepia and Ctn on treated carbon paper**

The C 1s spectra of Sepia and Ctn on TCP consist of six peaks: aromatic carbon C=C at 284.2 eV, aliphatic carbon (C–C) at 285.1 eV, carbon-nitrogen and ether (C–N, C–O–C) at 286.1 eV, hydroxyl (C–OH) at 287.4 eV, carbonyl (C=O) at 288.6 eV, and carboxyl (HO–C=O) at 289.2 eV along with a satellite peak (cyclic carbon structure) at 291.4 eV (Supplementary Figs. 5a and 5d). The intensity of the aforementioned peaks is higher than in the deconvoluted C 1s spectrum of the TCP, which confirms the successful deposition of Sepia and Ctn onto TCP. The N 1s spectrum indicates the presence of amino nitrogen atoms (C–NH at 400 eV) and heterocyclic nitrogen atoms (C–N=C at 401.6 eV) in Sepia on TCP and only the amino nitrogen atoms in Ctn on TCP (Supplementary Figs. 5b and 5e). The O 1s spectra of both Sepia and Ctn on TCP show similar signals to the deconvoluted O 1s spectrum of TCP (Supplementary Figs. 5c and 5f).


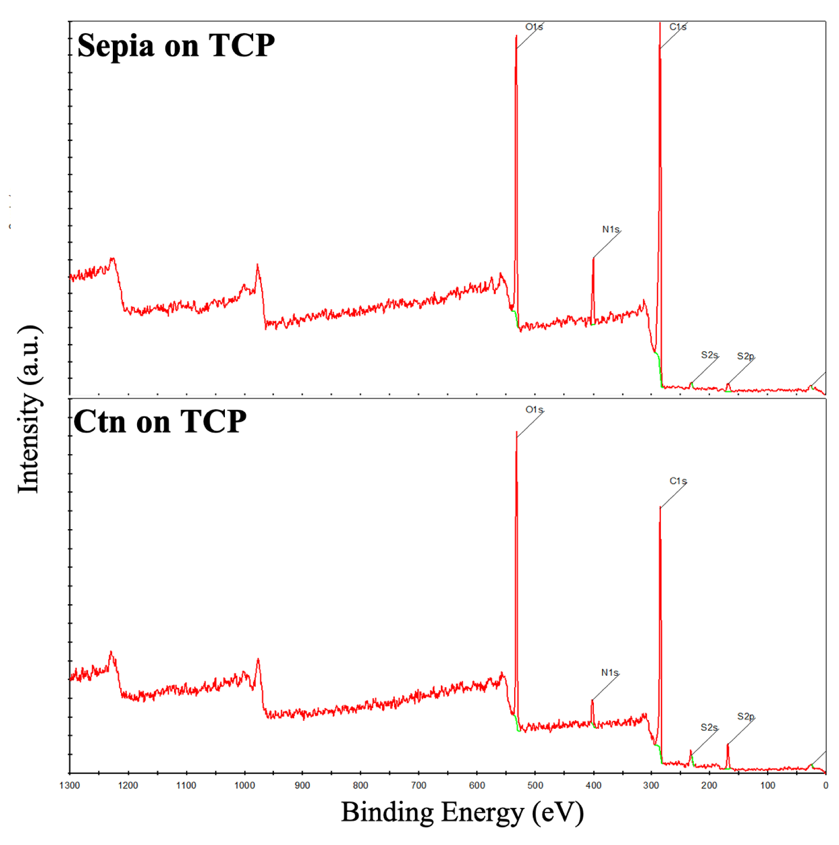


Supplementary Figure 4. Surface chemical composition of Sepia and Ctn on treated carbon paper (TCP). XPS survey spectra of Sepia and Ctn on treated carbon paper (TCP).


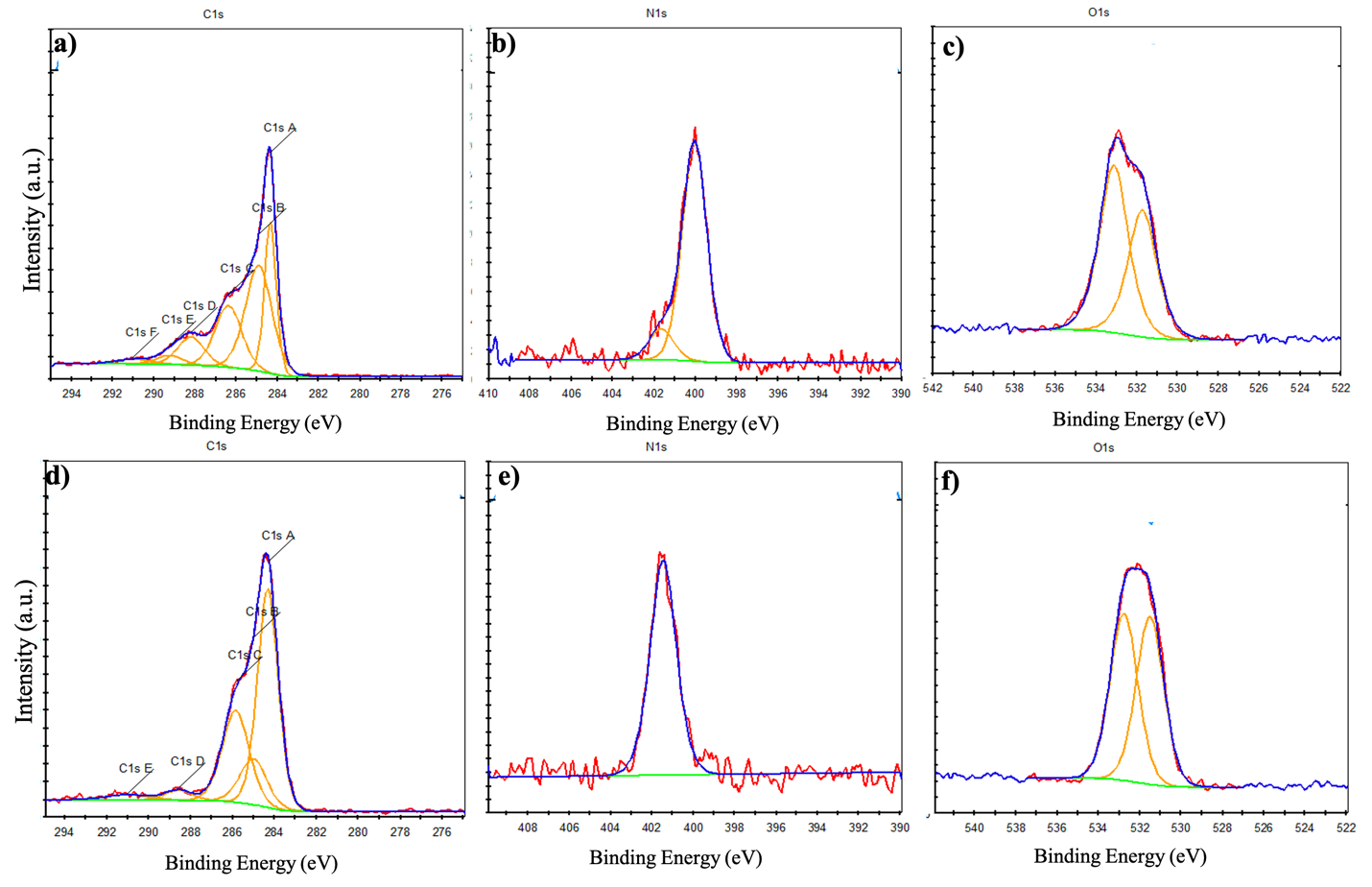


Supplementary Figure 5. High resolution surface chemical composition of Sepia and Ctn on treated carbon paper (TCP). Deconvoluted XPS spectra: (a) and (d) C 1s, (b) and (e) N 1s, and (c) and (f) O 1s for Sepia and Ctn on TCP, respectively.

Supplementary Figure 6. Electrochemical characterization of carbon paper (CP) and treated carbon paper (TCP) (without biosourced quinone material) in a 3-electrode cell setup. Cyclic voltammetry at different scan rates of (a) CP and (b) TCP, and (c) zoomed-in Nyquist plot for CP and TCP.

Supplementary Figure 7. Electrochemical characterization of carbon paper (CP), treated carbon paper (TCP), Sepia, Sepia/SP, and Sepia/r-GO on carbon paper and treated carbon paper in a 3-electrode cell setup. (a), (b), (d), and (f) show cyclic voltammetry at 5 mV s^-1^. (c), (e), and (g) are Nyquist plots in the frequency range 10^5^−10^−1^ Hz. Insets of (c) and (e) show CV of Sepia on carbon paper. (h) and (i) are the areal capacitance and the charge-transfer resistance (R_ch_) of different electrode materials evaluated from cyclic voltammetry at 5 mV s^-1^ and a zoomed-in Nyquist plot, respectively.


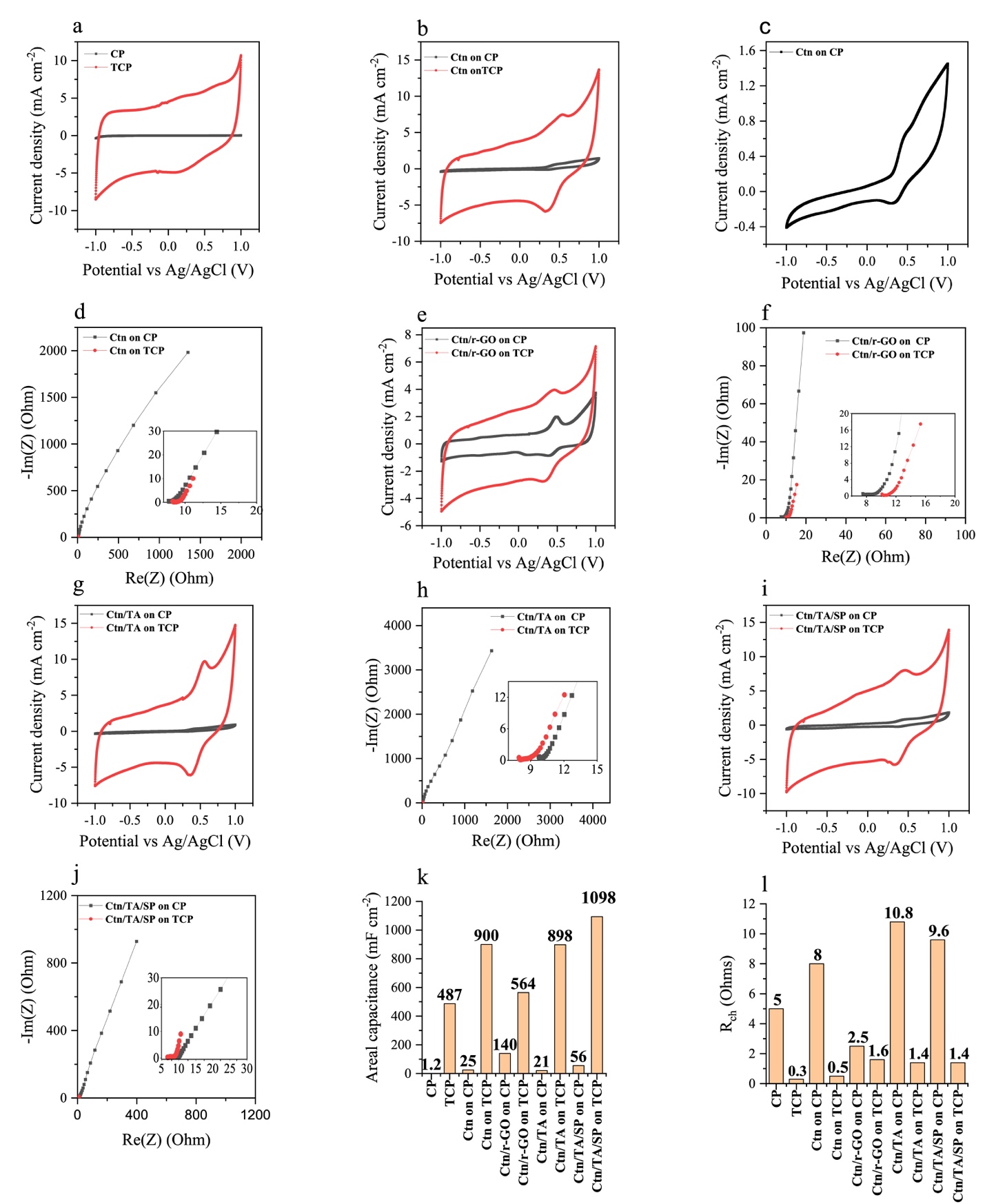


Supplementary Figure 8. Electrochemical characterization of carbon paper (CP) and treated carbon paper (TCP), catechin, catechin/r-GO, catechin/tannic acid, and catechin/tannic acid/SP on carbon paper (CP) and treated carbon paper (TCP) in a 3-electrode cell setup. (a), (b), (c), (e), (g), and (i) show cyclic voltammetry at 5 mV s^-1^. (d), (f), (h), and (j) are Nyquist plots in the frequency range 10^5^−10^−1^ Hz. Insets of (d), (f), (h), and (j) are zoomed-in Nyquist plots. (k) and (l) are the areal capacitance and the charge-transfer resistance of each electrode material evaluated from cyclic voltammetry at 5 mV s^-1^ and zoomed-in Nyquist plots.

**Supplementary Note 3: Evaluation of the Faradic and Non-Faradic Capacitance Contribution**

In pseudocapacitive materials, the current varies linearly with the potential scan rate, whereas in battery-like materials, it is proportional to the square root of the potential sweeping rate, according to ^7,8^

$$i\left( \nu\right)=a\nu^{b}$$

where *i* is the current and *ν* is the potential scan rate; *b* can be obtained from the slope of log *i* vs. log *ν*:

$$\log i=\log a+b \log\nu$$

A *b*-value of 0.5 indicates a diffusion-controlled electrode process, typical of battery-like materials, whereas a *b*-value of 1 indicates a fast, surface-confined redox process typical of pseudocapacitive materials.

Our Sepia and Ctn/TA on TCP electrode materials exhibit a b-value of about 0.85 and 0.80, respectively, i.e. they are pseudocapacitive electrode materials (Supplementary Fig. 9a).

We used the Trasatti method to evaluate faradaic vs. non-faradaic contributions^9^. We proceeded as follows:

1-We calculated **the areal capacitance (*C*)** from cyclic voltammetry curves at different scan rates, using:

$$C=\frac{S}{2 \nu\Delta V}$$

where *C* is the areal capacitance (mF cm^-2^), Δ*V* the potential window (in *V*), *S* the area enclosed by corresponding cyclic voltammograms (mAV cm^-2^), and *ν* the scan rate (V s^-1^).

2-We assumed that ion diffusion follows the Cottrell equation (semi-infinite diffusion); the **maximum areal capacitance (*C*_T_)** can be extracted from the linear relation between the reciprocal of the calculated areal capacitance (*C*^-1^) and the square root of scan rates (*ν*^1/2^):

$$C^{-1}=constant \nu^{1/2}+C_{T}^{-1}$$

where *C*_T_ is the sum of electrical double-layer capacitance (*C*_EDL_) and pseudocapacitance (*C*_PC_) (Supplementary Fig. 9b).^10^

3-We evaluated the **maximum EDLC and maximum PC** by plotting the linear relation between the calculated areal capacitances (*C*) vs. the reciprocal of square root of scan rates (*ν*^-1/2^) assuming semi-infinite diffusion:

$$C=constant \nu^{-1/2}+C_{EDL}$$

where ***C*_EDL_ is the maximum electrical double-layer capacitance** evaluated from the linear fitting and extrapolating the fitting line to y-axis (Supplementary Fig. 9c). The **maximum pseudocapacitance (*C*_PS_)** can be evaluated by subtracting *C*_EDL_ from *C*_T_.

4-Evaluting the percentage of capacitance contribution

$$C_{EDL}\%=\frac{C_{EDL}}{C_{T}}\times100$$

$$C_{PC}\%=\frac{C_{PC}}{C_{T}}\times100$$

In our case, *C*_T_, *C*_EDL_, and *C*_PC_ are 1443, 1116, 1062, 902, 381, and 214 mF/cm^2^ for Sepia and Ctn/TA on TCP, respectively. This means that the non-faradaic capacitance contributes about 73% and 85% while the pseudocapacitance contributes about 27% and 15% of the total capacitance for Sepia and Ctn/TA on TCP, respectively.


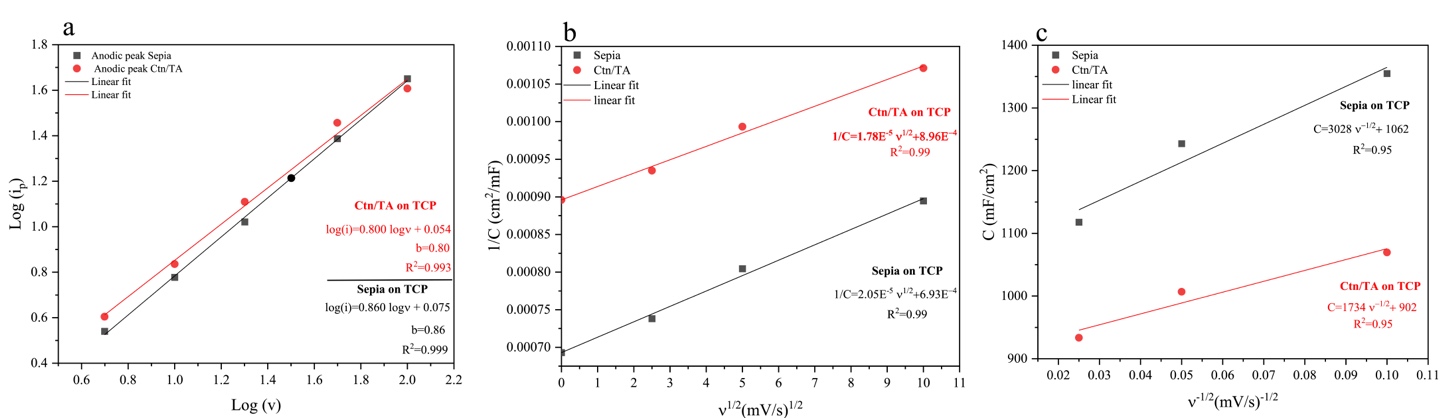


Supplementary Figure 9. Evaluation of the Faradic and Non-Faradic Capacitance Contribution. (a) Log(*i*) vs. log (*ν*) plot of the anodic peak currents extracted from the cyclic voltammograms in Fig.2 and S5. (b) Plot of reciprocal of areal capacitance (*C*^-1^) vs. square root of the scan rate (*ν*^1/2^). (c) Plots of areal capacitance (*C*) vs. reciprocal of square root of the scan rate (*ν*^-1/2^). The solid lines are linear fitting of data points, and the linear fit equation with root mean square is shown in the inset.

**Supplementary Note 4: Sepia and Catechin with Additives**

In general, biosourced quinone-based materials feature low electronic conductivity and high contact resistance on carbon. Sepia and Ctn/TA on CP feature capacitance and charge-transfer resistance of about 38 and 21 mF cm^-2^ and 4 and 11 ohms, respectively, as evaluated from cyclic voltammetry at 5 mV s^-1^ and Nyquist plots (Supplementary Figs. 7h, 7i, 8k, and 8l). So, we decided to explore the mixing of quinone-based materials (Sepia and catechin) with conductive additives (SP and r-GO).

Initially, we performed SEM characterization of the samples. SEM images revealed a network of spherical Sepia aggregates connected through r-GO flakes (Supplementary Fig. 10a). On the other hand, Ctn/r-GO samples showed segregation of Ctn and r-GO flakes (Supplementary Fig. 10b). SEM images of Sepia/SP exhibited a compact composite structure compared to that of bare Sepia or Sepia/r-GO composites (Supplementary Fig. 10c). SEM images of stained-Ctn/TA/SP exhibited geometric micrometric structures (inset of Supplementary Fig. 10d). Higher magnification images and lateral investigation also revealed isolated Ag particles on carbon fibers (Supplementary Fig. 10d).

8:2 w/w, 7:1:2 w/w, 5:5 w/w, and 5:5 w/w were the weight ratios for Sepia/SP, Ctn/TA/SP, Sepia/r-GO and Ctn/r-GO, respectively, resulting in the highest capacitance, i.e., 122, 56, 423, and 140 mF cm^-2^, and lowest charge-transfer resistance, i.e., 3.0, 9.6, 1.2, and 2.5 ohms cm^-2^ (Supplementary Figs. 7h, 8k, 11, and 12).


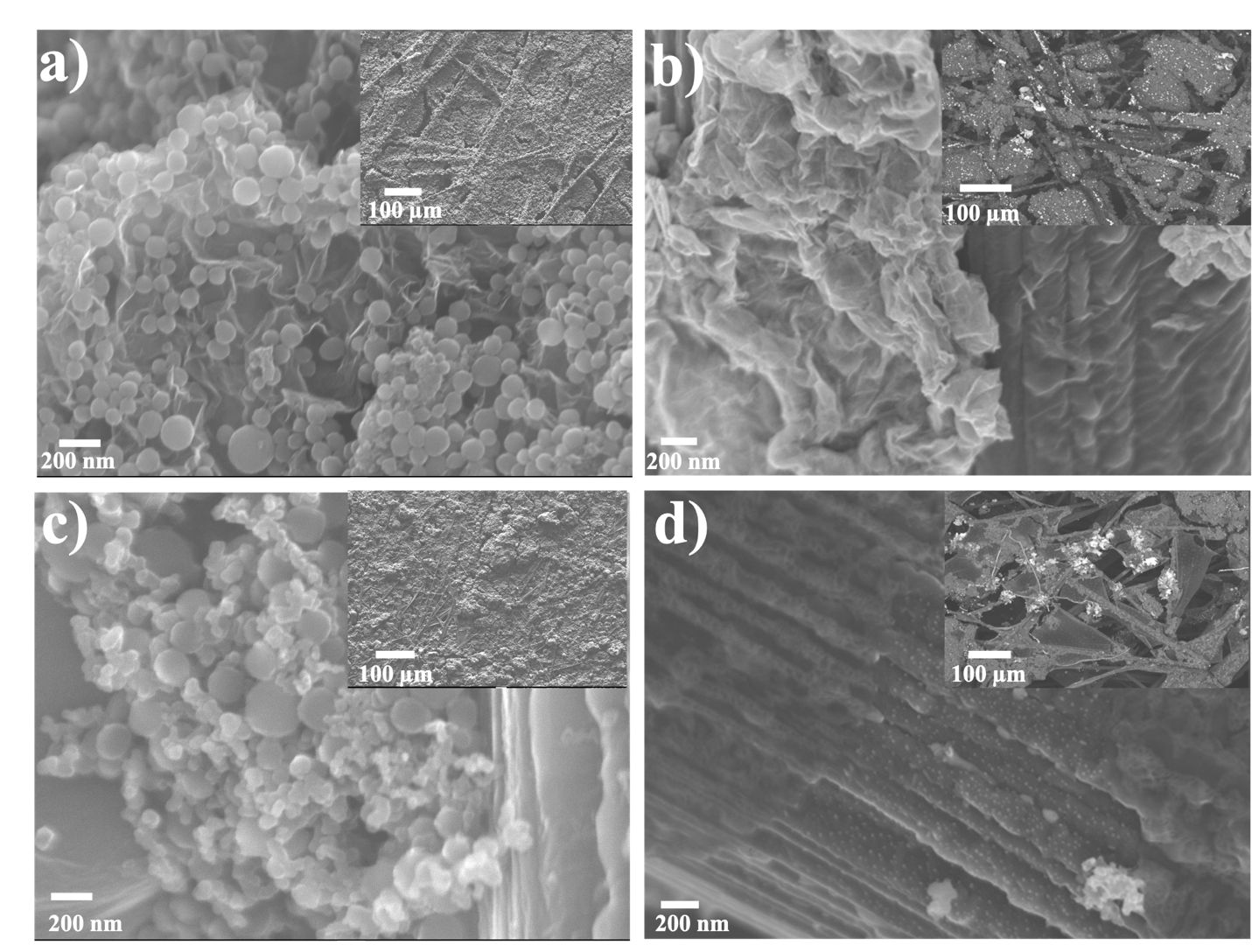


Supplementary Figure 10. Morphological characterization of Sepia and Catechin with additives. SEM images: (a) Sepia/r-GO, (b) silver-stained catechin/r-GO, (c) Sepia/SP, and (d) silver-stained catechin/tannic acid/SP on carbon paper (tilted samples). Inset figures are top views of the samples.

Supplementary Figure 11. Electrochemical characterization of Sepia/SP and Sepia/r-GO of different weight ratios on carbon paper in a 3-electrode cell setup. (a) and (c) are cyclic voltammetries at 5 mV s^-1^. (b) and (d) are Nyquist plots in the frequency range 10^5^−10^−1^ Hz.

Supplementary Figure 12. Electrochemical characterization of catechin/SP and catechin/r-GO of different weight ratios on carbon paper in a 3-electrode cell setup. (a) and (c) are cyclic voltammetries at 5 mV s^-1^. (b) and (d) are Nyquist plots in the frequency range 10^5^−10^−1^ Hz.

Supplementary Figure 13. Electrochemical characterization of symmetric supercapacitors based on TCP, Sepia, and Ctn/TA on TCP. Cyclic voltammetry at different potential windows at 20 mV s^-1^ scan rate. (a) TCP, (b) Sepia on TCP, and (c) Ctn/TA on TCP. (d) Capacitance retention and Coulombic efficiency of TCP-symmetric supercapacitor for 10 000 cycles of galvanostatic charge/discharge at 10 A g^-1^.


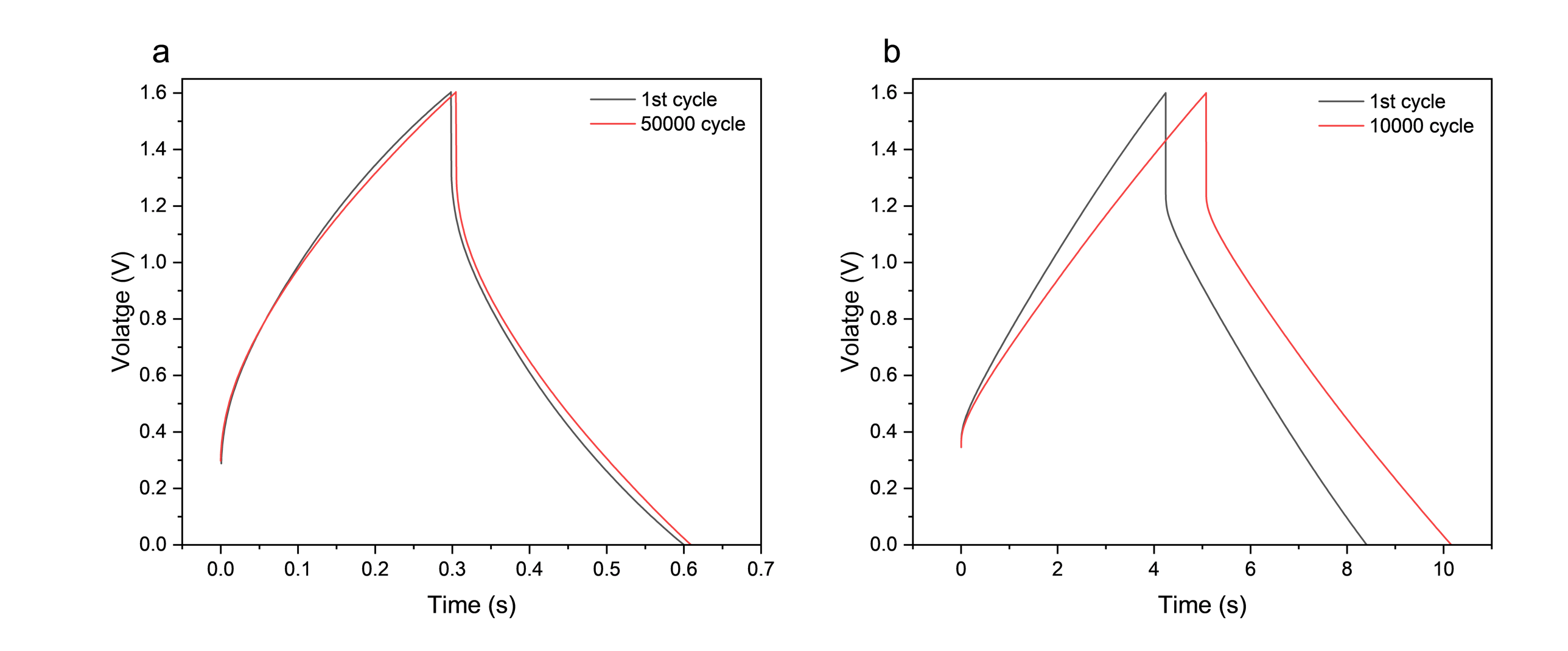


Supplementary Figure 14. Electrochemical characterization of symmetric supercapacitors based on Sepia and Ctn/TA. Galvanostatic charge and discharge curves at 10 A g^−1^ for symmetric supercapacitors based on a) Sepia on TCP and b) Ctn/TA on TCP.


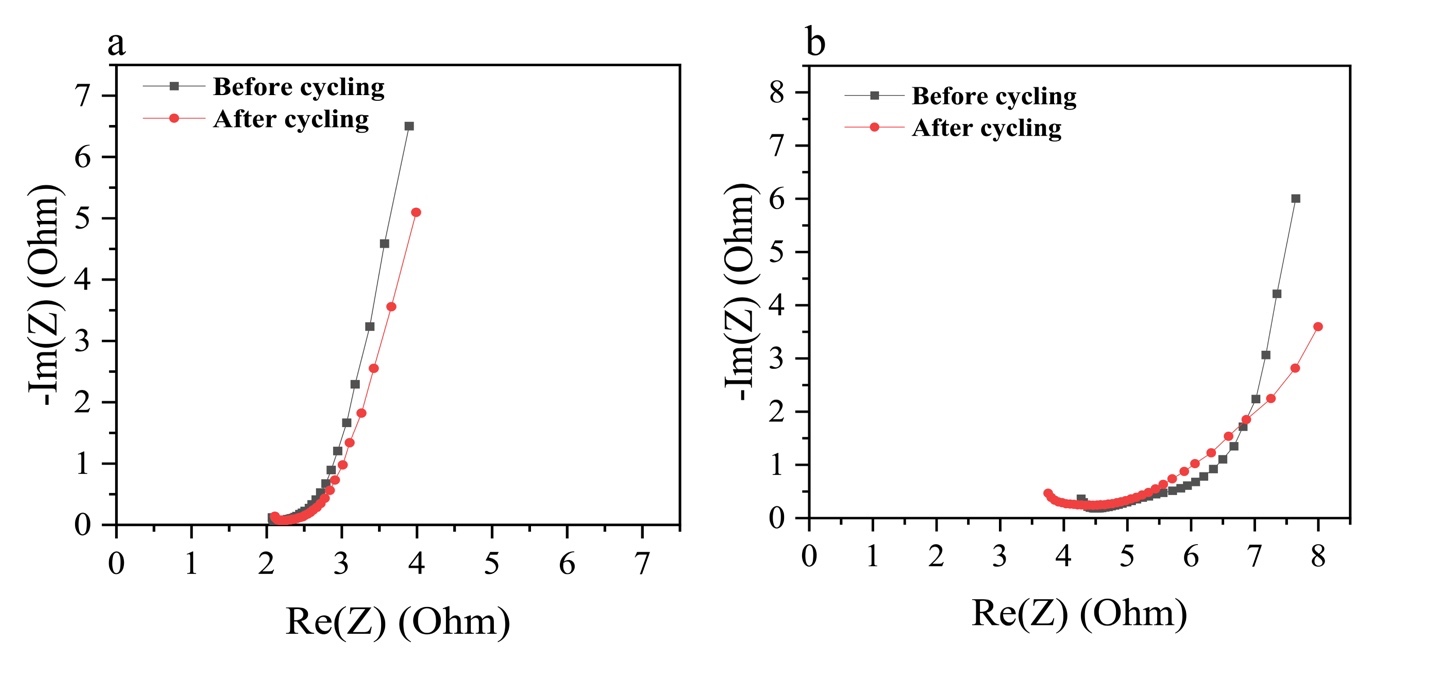


Supplementary Figure 15. Electrochemical impedance spectroscopy characterization of symmetric supercapacitors based on Sepia and Ctn/TA. Nyquist plot in the frequency range 10^5^–10^−1^ Hz for (a) Sepia and (b) Ctn/TA on TCP.

Supplementary Table 1. State-of-the-art of organic biosourced and inorganic materials as electrode materials for supercapacitors in the literature (see abbreviations below).

| **Electrode Material** | **Electrolyte** | **Performance** | | | | **Capacitance Retention** | **Ref** |
| --- | --- | --- | --- | --- | --- | --- | --- |
|  |  | **Capacitance** | **Charging voltage** | **Max. energy density (Wh kg^-1^)** | **Max. power density**  **(W kg^-1^)** |  |  |
| **Quinone-based electrodes** | | | | | | | |
| AQ/HP CNTs | 1M H_2_SO_4_ | 710 F g^-1^ at 1 A g^-1^ | 0.7 V | NA | NA | 96.45% after 1 000 cycles at 10 A g^-1^ | ^11^ |
| 2,6 dibromo-AQ/ carbon electrode | 0.5M LiClO_4_/acetonitrile | 650 F g^-1^ at 5 mV s^-1^ | 3 V | 45.50 | 21 400 | 85% after  1 000 cycles | ^12^ |
| AQ on modified AC | 0.1M H_2_SO_4_ | 195 F g^−1^ | 1 V | NA | NA | 94% after  10 000 cycles at 10 A g^-1^ | ^13^ |
| 2,5-dimethoxy-1,4-benzoquinone on graphene | 1M H_2_SO_4_ | 650 F g^−1^ at 5 mV s^−1^ | 0.9 V | NA | NA | 99% after 25 000 cycles at 50 mV s^−1^ | ^14^ |
| 2,5-dihydroxy-1,4-benzoquinone on reduced graphene oxide - Ti_3_C2T_x_ as asymmetric device | 3M H_2_SO_4_ | 90 F g^−1^ at 2 mV s^−1^ | 1.4 V | 2.9 kW kg^−1^ | 40 W h kg^−1^ | 90% after10 000 cycles | ^15^ |
| Benzoquinone and dopamine on graphite sheet | 1M H_2_SO_4_ | 399 F g^−1^ at 5 mV s^−1^ | 1 V | NA | NA | 50% after1 000 cycles | ^16^ |
| Carbonization of benzoquinone-amine polymers | 1M H_2_SO_4_ | 360 F g^-1^ at 0.5 A g^-1^ | 1.2 V | 18.20 | 30 000 | 90% after  100 000 cycles at 20 A g^-1^ | ^17^ |
| NQ-rGO/CF | PVA/KOH | 223 F g^-1^ at 0.21 A g^-1^ (NQrGO/CF) | 2 V | 50.7 | 1 642 | 88% after 10 000 cycles | ^18^ |
| **Polydopamine-based electrodes** | | | | | | | |
| PDA - GO/poly(3,4-ethylenedioxythio-phene) (GO/PEDOT) hybrid | 0.1M LiClO_4_ | 126 F g^-1^ at 1 A g^-1^ | 0.9 V | NA | NA | 75% after  1 000 cycles | ^19^ |
| PDA - AC | H_2_SO_4_/ hydroquinone (HQ) | 557 F g^−1^  at 2 A g^-1^ | 1 V | 19.36 | 25 000 | 98% after  10 000 cycles at 2 A g^-1^ | ^20^ |
| PDA ‐ rGO | 6M KOH | 200 F g^−1^ at 1 A g^−1^ | 1 V | NA | NA | 99% after  10 000 cycles at 2 A g^-1^ | ^21^ |
| rGO/PDA-carbon cloth | 1M H_2_SO_4_ | 1 209 mF cm^-2^ at 1 mA cm^−2^ | 0.8 V | NA | NA | NA | ^22^ |
| PDA-based N-doped. carbon spheres+ Co_x_Ni_1_- x(OH)_2_ | 6M KOH | 2 065 F g^−1^ at 1 A g^−1^ | 1.6 V | 37.00 | 8 000 | 64% after 5 000 cycles | ^23^ |
| PDA on oxygen-functionalized carbon cloth | PVA)-in-H_2_SO_4_ | 617 mF cm^−2^  at 2.2 mA cm^−2^ | 1.2 V | 11.70 | 6.400 | 81% after  10 000 cycles | ^24^ |
| **N-doped carbon electrodes** | | | | | | | |
| N-doped CF | KOH (6M) - pH > 14 | 236 F g^-1^ at  0.2 A g^-1^,  1045 F g^-1^ at  1 mV s^-1^ | 1 V | NA | NA | > 98% after  10 000 cycles at 20 A g^-1^, 84% after  5 000 cycles at 100 mV s^-1^ | ^25^ |
| N-doped CA | 0.5M H_2_SO_4_ | 426 F g^-1^ at 1 A g^-1^ | 1.6 V | NA | NA | 104% after  10 000 cycles | ^26^ |
| HP N-doped carbon via hydrothermal treatment and activation of lignin | 6M KOH and EMI-BF for cycling | 310 F g^-1^ in aqueous media, 140 F g^-1^ in  IL at 1 A g^-1^ | 1 V in aqueous media, 3V in IL | NA | NA | 98% after  20 000 cycles | ^27^ |
| N-doped porous carbon from cellulose with urea treatment | 1M H_2_SO_4_ | 160 F g^-1^ at 1 A g^-1^, 340 F g^-1^ at 5 mV s^-1^ | 0.8 V | NA | NA | 107% after  5 000 cycles | ^28^ |
| N-doped porous carbon from potato residue waste | 2M KOH | 255 F g^-1^ at 0.5 A g^-1^ | 1 V | NA | NA | 94 % after  5 000 cycles | ^29^ |
| N-doped porous carbon from cellulose via dissolving-gelling process | 1M H_2_SO_4_ | 389 F g^-1^ at  5 mV s^-1^, 225 F g^-1^  at 0.5 A g^-1^ | 0.8 V | NA | NA | >90 % after  5 000 cycles | ^30^ |
| N-doped porous carbon from chitosan | 6M KOH and KOH/PVA | 292 F g^-1^ at 2 mV s^-1^ for aqueous media, 192 F g^-1^ at 2 mV s^-1^ for solid state | 1 V in aqueous media | NA | NA | 92% after  10 000 cycles for the hydrogel device | ^31^ |
| N, O co-doped carbon nanofoam | 6M KOH | 367 F g^−1^ at 0.5 A g^−1^ | 1 V | 24.6 | 400 | 96.2 % after 10 000 cycles at 10 A g^-1^ | ^32^ |
| N-doped porous carbon N-rich sword bean shells | 6M KOH | 264 F g^−1^ at 1 A g^−1^ |  | 12.5 | 5 000 | 100 % after 10 000 cycles at 1 A g^-1^ | ^33^ |
| **Electrode through carbonization of organic precursor** | | | | | | | |
| Pyrolysis of corn husk | 6M KOH and 1M Na_2_SO_4_ | 356 F g^-1^ at 1 A g^-1^ | 1 V | NA | NA | 95% after  2 500 cycles | ^34^ |
| Carbonized and freeze-dried bagasse aerogel | PVA/KOH gel electrolyte | 142 F g^-1^ at 0.5 A g^-1^ | 1 V | NA | NA | 94 % after  5 000 cycles | ^35^ |
| Carbonized Cellulose nanofibril/GO composite aerogels | 1M H_2_SO_4_ | 398 F g^-1^ at 0.5 A g^-1^ | 0.9 V | NA | NA | 99.86% after 10 000 cycles | ^36^ |
| Carbon spheres from hemp stem hemicellulose carbonization | 6M KOH | 318 F g^-1^ at 0.1 A g^-1^ | 1 V | NA | NA | 96 % after  10 000 cycles | ^37^ |
| Carbonized cellulose | 5M KCL | 115 F g^-1^ at 5 mV s^-1^ and 1 A g^-1^ | 0.8 V | NA | NA | 87 % after  3 000 cycles | ^38^ |
| Carbonized egg yolk | 1M H_2_SO­_4_ | 288 F g^-1^ at 30 A g^-1^ | 1 V | 12.70 | 6 000 | 94.5% after  10 000 cycles at 30 A g^-1^ | ^39^ |
| Carbonization of chitosan-amino acid gel | 6M KOH or 1M Na_2_SO_4_ | 478 F g^-1^ at 0.5 A g^-1^ | 1 V for KOH, 2V for Na_2_SO­_4_ | 30.10 | 7 251 | 100% after 100 000 cycles | ^40^ |
| **Melanin-based electrodes** | | | | | | | |
| Sigma melanin/CP | 0.25 M NH_4_CH_3_COOH | 5.6 mF cm^-2^ at  5 mV s^-1^ | 0.8 V | 0.31 | 13 100 | 75% after  1 000 cycles | ^41^ |
| Synthetic melanin/CP | 0.25 M Na CH_3_COO | 5.6 mF cm^-2^ at  5 mV s^-1^ | 0.4 V | 0.02 | 5900 | 96% after  5 000 cycles | ^42,43^ |
| MnO_2_-Synthetic melanin | 6 M KOH | 545 F g^−1^ at 0.5 A g^−1^ | 1.7 V | 27.00 | 3 528 | 87% after 3 000 cycles | ^44^ |
| Sepia/N,S GCQDs on CP | 0.5 M Na_2_SO_4_ | 180 mF cm^-2^ at 5 mV s^-1^ | 1 V | 1.38 | 12 750 | 92% after  10 000 cycles | ^45^ |
| **Tannin-based electrodes** | | | | | | | |
| TA and Fe3+ on fiber- glass | 1M LiCl and LiCl/PVA hydrogel | 423 mF cm^-2^  at 1 mA cm^-2^ for electrode,  70 mF cm^-2^ at  0.5 mA cm^-2^ for hydrogel | 2.4 V | NA | NA | >81% for electrode and >88% for hydrogel after 5 000 cycles | ^46^ |
| O-doped HP carbons from gallic acid | 1M H_2_SO_4_ | 277 F g^-1^ at 0.5 mV s^-1^, 196 F g^-1^ at 0.1 A g^-1^ | 0.8 V | NA | NA | 80% after  5 000 cycles | ^47^ |
| Calcinated TA-derived carbon | H_2_SO_4_/PVA hydrogel | 315 mF cm^-2^ at 1 mA cm^-2^ | 0.8 V | NA | NA | 93% after  10 000 cycles | ^48^ |
| TA - graphene hydrogel | H_2_SO_4_/PVA hydrogel | 533 F g^-1^ at 0.5 A g^-1^ | 1 V | NA | NA | 84% after  8 000 cycles | ^49^ |
| PANI/TA/rGO | H_2_SO_4_/PVA hydrogel | 269 F g^-1^ at 10 mV s^-1^ | 1.6 V | NA | NA | 80% after 200 cycles | ^50^ |
| Mimosa tannin mechanosynthesis and pyrolysis | 1M H_2_SO_4_ | 40 F g^-1^ at 5 mV s^-1^, 37 F g^-1^ at 0.2 A g^-1^ | 1 V | NA | NA | >94% after  10 000 cycles | ^51^ |
| Bark tannins/polypyrrole composite on gold or carbonized wood | 0.1M HClO_4_ | 370 F g^-1^ at 0.5 A g^-1^ for gold, 4.6 F cm^-2^ at 0.5 mA cm^-2^ for carbonized wood | 0.6 V | NA | NA | NA | ^52^ |
| Activated mesoporous carbon derived from mimosa tannin | 1M H_2_SO_4_  & 1M TEABF_4_/  ACN | 40 F g^−1^ | 1 V (1M H_2_SO_4_ )  2.7 V (1M TEABF_4_/  ACN) | 5.00 | 44 | 95% after 10 000 cycles (1M H_2_SO_4_) | ^53^ |
| AC from pine tannin | 1M H_2_SO_4_ | 232 F g^-1^ at 0.5 A g^-1^ | 0.9V | 6.70 | 1 000 | 94% after 10 000 cycles | ^54^ |
| **Lignin-based electrodes** | | | | | | | |
| Al/AC/lignin-MnO_2_ | PVA/H_3_PO_4_ | 170 F g^-1^  at 10 mV s^-1^ | 1 V | NA | NA | 97.5% after  2 000 cycles | ^55^ |
| Al/lignin–NiWO_4_ | PVA/H_3_PO_4_ | 17 mF cm^-2^  at 0.1 A g^-1^ | 1 V | NA | NA | 84% after  2 000 cycles | ^56^ |
| Pre-crossed-linked lignin gel | 6M KOH | 90 F g^-1^ | 1.2 V | NA | NA | NA | ^57^ |
| Lignin-derived HP carbon | 1M H_2_SO_4_ | 165 F g^-1^ at 1mV s^-1^, 165 F g^-1^ at 0.05 A g^-1^ | 1.3 V | NA | NA | > 97% after  5 000 cycles | ^58^ |
| Lignin in graphene cage on gold | 0.1M HClO_4_ | 211 F g^-1^ at 1 A g^-1^ | 0.8 V | NA | NA | 88% after  15 000 cycles | ^59^ |
| Lignocellulose (from citrus peel) based AC | KOH-PVA | 565 F g^-1^ | 1.1 V | 24.50 | 802 | 98% after  10 000 cycles | ^60^ |
| **Inorganic and metal-based electrodes** | | | | | | | |
| MoS_2_/graphene oxide/meso-MnO_2_ nanocomposite | 1M H_2_SO_4_ | 980 F g^-1^ at 1 mV s^-1^, 980 F g^-1^ at 0.5 A g^-1^ | 0.3 V | NA | NA | > 85% after  5 000 cycles | ^61^ |
| Mn_3_O_4_ nanoflakes/rGO composite | 1M Na_2_SO_4_ | 351 F g^-1^ at 0.5 A g^-1^ | 1 V | NA | NA | 80 % after  10 000 cycles | ^62^ |
| CoNi_2_S_4_/graphene nanocomposite | 3M KOH | 2 000 F g^-1^ at 1 A g^-1^ | 0.5 V | NA | NA | 75% after 2 000 cycles | ^63^ |
| Ni–Co-layered double hydroxide/PANI/BC | 2M KOH | 1 600 F g^-1^ at 1 A g^-1^ | 1.6 V | NA | NA | 84% after  5 000 cycles | ^64^ |
| NiCo_2_S_4_/carbon and PC from cellulose | 2M KOH | 154 F g^-1^ at 1 A g^-1^ | 1.6 V | NA | NA | 97% after  10 000 cycles | ^65^ |
| TiO_2_/ AC from oleander seeds | 1 M Na2SO4 | 214 F g^-1^ at 1  A g^-1^ | 1.1 V | 27.40 | 4 000 | 92% after  5 000 cycles | ^66^ |
| **Other electrodes** | | | | | | | |
| Juglone/PPy/CF | 1M H_2_SO_4_ | 1.7 mF cm^-1^  at 5 mV s^-1^ | 0.8 V | NA | NA | 95% after  1 000 cycles | ^67^ |
| Pyrene-4,5 dione/carbon  onions | 1M H_2_SO_4_ | 264 F g^-1^ at 5 mV s^-1^ | 0.8 V | 4.5 | NA | 97% after  10 000 cycles at 1.3 A g^-1^ | ^68^ |
| Emodin (6-methyl-1,3,8 trihydroxy-AQ)/PPy/CF | 0.5M H_2_SO_4_ | 9.5 mF cm^-1^  at 5 mV s^-1^ | 1.1 V | 1 mWh cm^-3^ | 579 mW cm^-3^ | 83% after  2 000 cycles | ^69^ |
| 1-amino-AQ on Ti_3_C_2_T_x_ MXene | 3M H_2_SO_4_ | 300 F g^-1^ at 5 mV s^-1^ | 0.8 V | NA | NA | NA | ^70^ |
| 3,4,9,10-perylenetetracarboxylic acid/rGO | 1M H_2_SO_4_ | 422.7 F g^-1^  at 10 mV s^-1^ | 1.1 V | 14.00 | 4 894 | 82% after  3 000 cycles at 3 A g^–1^ | ^71^ |
| AC from Kelp | Sodium alginate gel | 227 F g^-1^ at 0.1 A g^-1^ | 1 V | 8.00 | 5 000 | 98.5% after  10 000 cycles at 5 A g^-1^ | ^72^ |
| MnO_2_/carbon cloth | 0.1 M Na_2_SO_4_ | 684 F g^-1^ at 2 A g^-1^ | 0.8 V | 46.50 | 45 000 | 94% after  1 000 cycles | ^73^ |
| CNT/BC | [EMIM]  [NTf_2_] | 47 F g^-1^ at 100 mV s^-1^ | 3 V | NA | NA | > 99.5% after 5 000 cycles at 10 A.g^-1^ | ^74^ |
| BC/PANI | 1M H_2_SO_4_ | 273 F g^-1^ at 0.2 A g^-1^ | 1 V | NA | NA | 94% after  1 000 cycles | ^75^ |
| Hierarchical mesoporous yolk–shell-structured carbon nanospheres | 1M KOH | 159 F g^-1^ at 10 mV s^-1^ | 1.4 V | NA | NA | NA | ^76^ |
| PANI/cellulose films | 1M H_2_SO_4_ | 160 F g^-1^ at 0.1 A g^-1^ | 1 V | NA | NA | 81% after  1 000 cycles | ^77^ |
| PHATN on CP | 6M KOH | 689 F g^-1^ at 0.1 A g^-1^ | 1.2 V | NA | NA | 100% capacity retention after 50 000 cycles at 50 A g^-1^ | ^78^ |
| **This work** | | | | | | | |
| Sepia on TCP | 0.5 M Na_2_SO_4_ | 1 355 mF cm^-2^ ~ 452 F g^-1^ at 5 mV s^-1^ | 1.6 V | 20.00 | 46 000 | 100% after  50 000 cycles at 10 A g^-1^ |  |
| Ctn/TA on TCP | 0.5 M Na_2_SO_4_ | 898 mF cm^-2^ ~ 300 F g^-1^ at 5 mV s^-1^ | 1.6 V | 23.00 | 26 000 | 100% after  10 000 cycles at 10 A g^-1^ |  |

Abbreviations: Q: quinone, AQ: anthraquinone, HQ: hydroquinone, NQ: naphthoquinone, AC: activated carbon, CNTs: carbon nanotubes, CP: carbon paper, CF: carbon fiber, HP: hierarchical porous, PC: porous carbon, CA: carbon aerogel, GO: graphene oxide, rGO: reduced GO, PDA: polydopamine, ppy: polypyrrole, BC: bacterial nanocellulose, N,S GCQDs: nitrogen and sulphur-doped graphitic carbon quantum dots, PHATN: perylene diimide and hexaazatrinaphthylene, TA: tannic acid, IL: ionic liquid, and TCP: treated carbon paper.

Supplementary Table 2. Summary of BET results indicating the structural properties of carbon paper (CP) and treated carbon paper (TCP).

| **Sample** | ***S*_BET_ (m^2^ g^-1^)** | ***S*_micro_ (m^2^ g^-1^)** | ***S*_micro_/*S*_BET_** | ***V*_total_ (cm^3^ g^-1^)** | ***V*_micro_ (cm^3^ g^-1^)** | ***V*_micro_/*V*_total_** | ***D* (nm)** |
| --- | --- | --- | --- | --- | --- | --- | --- |
| **CP** | 0.37 | 0.12 | 0.32 | $5\times{10}^{-3}$ | $6\times{10}^{-4}$ | 0.12 | 159 |
| **TCP** | 43.04 | 38.05 | 0.88 | $5\times{10}^{-2}$ | $2\times{10}^{-3}$ | 0.04 | 5.1 |

*S*_BET_: BET surface area; *S*_Micro_: micropore surface area; *V*_micro_: micropore volume; *V*_total_: total pore volume; and *D*: average pore diameter.

Supplementary Table 3. Identification of chemical bonding from high-resolution XPS scans for carbon paper and treated carbon paper.

| Group Name | Binding Energy (eV) | Identification | Atomic % | |
| --- | --- | --- | --- | --- |
|  |  |  | CP | TCP |
| P - 2p | 131.60 | P-C | -- | 0.5 |
|  | 133.30 | P-N | -- | 0.2 |
| S - 2p | 168.22 | C-SO_x_-C | -- | 1 |
| C - 1s | 285.10 | element total | 94.6 | 79.8 |
|  | 284.30 | C=C | 78.6 | 31.8 |
|  | 284.80 | C-C | 10.2 | 29.5 |
|  | 286.10 | C-O | 0.7 | 5.5 |
|  | 287.80 | C=O | 1.2 | 1.9 |
|  | 289.00 | O-C=O | 0.8 | 5.1 |
|  | 291.00 | π→π* C=C | 3.7 | 3.9 |
| N - 1s | 400.70 | element total | -- | 8.3 |
|  | 399.90 | N-(C-O)-N | -- | 3.2 |
|  | 401.90 | N-C | -- | 1.2 |
|  | 406.40 | planar NO_2_ | -- | 0.5 |
| O - 1s | 533.10 | element total | 5.1 | 10.3 |
|  | 531.80 | -OH | -- |  |
|  | 533.30 | COOH | 1.8 | 7.7 |
|  | 534.60 | water peak | 0.3 | -- |

**Supplementary References**

1 Rahmanian, S., Suraya, A. R., Zahari, R. & Zainudin, E. S. Synthesis of vertically aligned carbon nanotubes on carbon fiber. *Appl. Surf. Sci.* **271**, 424-428, doi:<https://doi.org/10.1016/j.apsusc.2013.01.207> (2013).

2 Peng, J. *et al.* Graphene quantum dots derived from carbon fibers. *Nano Lett.* **12**, 844-849 (2012).

3 Gu, W., Sevilla, M., Magasinski, A., Fuertes, A. B. & Yushin, G. Sulfur-containing activated carbons with greatly reduced content of bottle neck pores for double-layer capacitors: a case study for pseudocapacitance detection. *Energy & Environmental Science* **6**, 2465-2476, doi:10.1039/C3EE41182F (2013).

4 Song, W. *et al.* Graphene ultracapacitors: structural impacts. *Physical Chemistry Chemical Physics* **15**, 4799-4803, doi:10.1039/C3CP50516B (2013).

5 Yang, Y. *et al.* “Protrusions” or “holes” in graphene: which is the better choice for sodium ion storage? *Energy & Environmental Science* **10**, 979-986, doi:10.1039/C7EE00329C (2017).

6 Song, W. *et al.* Tuning the Double Layer of Graphene Oxide through Phosphorus Doping for Enhanced Supercapacitance. *ACS Energy Letters* **2**, 1144-1149, doi:10.1021/acsenergylett.7b00275 (2017).

7 Liu, J. *et al.* Advanced Energy Storage Devices: Basic Principles, Analytical Methods, and Rational Materials Design. *Advanced Science* **5**, 1700322, doi:<https://doi.org/10.1002/advs.201700322> (2018).

8 Forghani, M. & Donne, S. W. Method Comparison for Deconvoluting Capacitive and Pseudo-Capacitive Contributions to Electrochemical Capacitor Electrode Behavior. *Journal of The Electrochemical Society* **165**, A664-A673, doi:10.1149/2.0931803jes (2018).

9 Ardizzone, S., Fregonara, G. & Trasatti, S. “Inner” and “outer” active surface of RuO2 electrodes. *Electrochimica Acta* **35**, 263-267, doi:<https://doi.org/10.1016/0013-4686(90)85068-X> (1990).

10 Lee, Y.-H., Chang, K.-H. & Hu, C.-C. Differentiate the pseudocapacitance and double-layer capacitance contributions for nitrogen-doped reduced graphene oxide in acidic and alkaline electrolytes. *Journal of Power Sources* **227**, 300-308, doi:<https://doi.org/10.1016/j.jpowsour.2012.11.026> (2013).

11 Chen, X. *et al.* Anthraquinone on Porous Carbon Nanotubes with Improved Supercapacitor Performance. *The Journal of Physical Chemistry C* **118**, 8262-8270, doi:10.1021/jp5009626 (2014).

12 Zhou, Y. *et al.* Polyanthraquinone-based nanostructured electrode material capable of high-performance pseudocapacitive energy storage in aprotic electrolyte. *Nano Energy* **15**, 654-661, doi:<https://doi.org/10.1016/j.nanoen.2015.05.029> (2015).

13 Pognon, G., Brousse, T., Demarconnay, L. & Bélanger, D. Performance and stability of electrochemical capacitor based on anthraquinone modified activated carbon. *J. Power Sources* **196**, 4117-4122, doi:<https://doi.org/10.1016/j.jpowsour.2010.09.097> (2011).

14 Boota, M., Chen, C., Bécuwe, M., Miao, L. & Gogotsi, Y. Pseudocapacitance and excellent cyclability of 2,5-dimethoxy-1,4-benzoquinone on graphene. *Energy & Environmental Science* **9**, 2586-2594, doi:10.1039/C6EE00793G (2016).

15 Boota, M., Chen, C., Van Aken, K. L., Jiang, J. & Gogotsi, Y. Organic-inorganic all-pseudocapacitive asymmetric energy storage devices. *Nano Energy* **65**, 104022, doi:<https://doi.org/10.1016/j.nanoen.2019.104022> (2019).

16 Ega, S. P., Biradar, M. R., Srinivasan, P. & Bhosale, S. V. Designing quinone-dopamine-based conjugates as six electron system for high-performance hybrid electrode. *Electrochimica Acta* **357**, 136835, doi:<https://doi.org/10.1016/j.electacta.2020.136835> (2020).

17 Song, Z. *et al.* A universal strategy to obtain highly redox-active porous carbons for efficient energy storage. *Journal of Materials Chemistry A* **8**, 3717-3725, doi:10.1039/C9TA13520K (2020).

18 Zhou, C., Gao, T., Liu, Q., Wang, Y. & Xiao, D. Preparation of quinone modified graphene-based fiber electrodes and its application in flexible asymmetrical supercapacitor. *Electrochimica Acta* **336**, 135628, doi:<https://doi.org/10.1016/j.electacta.2020.135628> (2020).

19 Cha, I. *et al.* Facile electrochemical synthesis of polydopamine-incorporated graphene oxide/PEDOT hybrid thin films for pseudocapacitive behaviors. *Synth. Met.* **195**, 162-166, doi:<https://doi.org/10.1016/j.synthmet.2014.05.019> (2014).

20 Zhang, Z. J. *et al.* Highly boosting the supercapacitor performance by polydopamine-induced surface modification of carbon materials and use of hydroquinone as an electrolyte additive. *Electrochimica Acta* **339**, 135940, doi:<https://doi.org/10.1016/j.electacta.2020.135940> (2020).

21 Dong, S. *et al.* Polydopamine-Modified Reduced Graphene Oxides as a Capable Electrode for High-Performance Supercapacitor. *ChemistrySelect* **4**, 2711-2715, doi:10.1002/slct.201900242 (2019).

22 Chen, R. *et al.* A multifunctional interface design on cellulose substrate enables high performance flexible all-solid-state supercapacitors. *Energy Storage Materials* **32**, 208-215, doi:<https://doi.org/10.1016/j.ensm.2020.07.030> (2020).

23 Jing, L. *et al.* Rapid and large-scale synthesis of polydopamine based N-doped carbon spheres@CoxNi1-x(OH)2 core-shell nanocomposites for high performance supercapacitors. *Journal of Alloys and Compounds* **854**, 157246, doi:<https://doi.org/10.1016/j.jallcom.2020.157246> (2021).

24 Moloudi, M. *et al.* Bioinspired polydopamine supported on oxygen-functionalized carbon cloth as a high-performance 1.2 V aqueous symmetric metal-free supercapacitor. *Journal of Materials Chemistry A*, doi:10.1039/D0TA12624A (2021).

25 Cai, J. *et al.* High-Performance Supercapacitor Electrode Materials from Cellulose-Derived Carbon Nanofibers. *ACS Applied Materials & Interfaces* **7**, 14946-14953, doi:10.1021/acsami.5b03757 (2015).

26 Gao, J. *et al.* Ethylenediamine-Catalyzed Preparation of Nitrogen-Doped Hierarchically Porous Carbon Aerogel under Hypersaline Condition for High-Performance Supercapacitors and Organic Solvent Absorbents. *Nanomaterials* **9**, doi:10.3390/nano9050771 (2019).

27 Zhang, L., You, T., Zhou, T., Zhou, X. & Xu, F. Interconnected Hierarchical Porous Carbon from Lignin-Derived Byproducts of Bioethanol Production for Ultra-High Performance Supercapacitors. *ACS Applied Materials & Interfaces* **8**, 13918-13925, doi:10.1021/acsami.6b02774 (2016).

28 Chen, Z. *et al.* Facile synthesis of cellulose-based carbon with tunable N content for potential supercapacitor application. *Carbohydrate Polymers* **170**, 107-116, doi:<https://doi.org/10.1016/j.carbpol.2017.04.063> (2017).

29 Ma, G. *et al.* Nitrogen-doped porous carbon derived from biomass waste for high-performance supercapacitor. *Bioresource Technology* **197**, 137-142, doi:<https://doi.org/10.1016/j.biortech.2015.07.100> (2015).

30 Hu, Y. *et al.* 3D hierarchical porous N-doped carbon aerogel from renewable cellulose: an attractive carbon for high-performance supercapacitor electrodes and CO2 adsorption. *RSC Advances* **6**, 15788-15795, doi:10.1039/C6RA00822D (2016).

31 Hao, P. *et al.* Graphene-based nitrogen self-doped hierarchical porous carbon aerogels derived from chitosan for high performance supercapacitors. *Nano Energy* **15**, 9-23, doi:<https://doi.org/10.1016/j.nanoen.2015.02.035> (2015).

32 Zhao, Y. *et al.* Nitrogen/oxygen co-doped carbon nanofoam derived from bamboo fungi for high-performance supercapacitors. *J. Power Sources* **479**, 228835, doi:<https://doi.org/10.1016/j.jpowsour.2020.228835> (2020).

33 Chen, T. *et al.* High energy density supercapacitors with hierarchical nitrogen-doped porous carbon as active material obtained from bio-waste. *Renewable Energy* **175**, 760-769, doi:<https://doi.org/10.1016/j.renene.2021.05.006> (2021).

34 Song, S. *et al.* Facile self-templating large scale preparation of biomass-derived 3D hierarchical porous carbon for advanced supercapacitors. *Journal of Materials Chemistry A* **3**, 18154-18162 (2015).

35 Hao, P. *et al.* Hierarchical porous carbon aerogel derived from bagasse for high performance supercapacitor electrode. *Nanoscale* **6**, 12120-12129, doi:10.1039/C4NR03574G (2014).

36 Yang, Q., Yang, J., Gao, Z., Li, B. & Xiong, C. Carbonized Cellulose Nanofibril/Graphene Oxide Composite Aerogels for High-Performance Supercapacitors. *ACS Applied Energy Materials* **3**, 1145-1151, doi:10.1021/acsaem.9b02195 (2020).

37 Wang, Y., Yang, R., Li, M. & Zhao, Z. Hydrothermal preparation of highly porous carbon spheres from hemp (Cannabis sativa L.) stem hemicellulose for use in energy-related applications. *Industrial Crops and Products* **65**, 216-226, doi:<https://doi.org/10.1016/j.indcrop.2014.12.008> (2015).

38 Jiang, L. *et al.* Cellulose-Derived Supercapacitors from the Carbonisation of Filter Paper. *ChemistryOpen* **4**, 586-589, doi:10.1002/open.201500150 (2015).

39 Xu, H., Wu, C., Wei, X. & Gao, S. Hierarchically porous carbon materials with controllable proportion of micropore area by dual-activator synthesis for high-performance supercapacitors. *Journal of Materials Chemistry A* **6**, 15340-15347, doi:10.1039/C8TA04777D (2018).

40 Yang, L., Wu, D., Wang, T. & Jia, D. B/N-Codoped Carbon Nanosheets Derived from the Self-Assembly of Chitosan–Amino Acid Gels for Greatly Improved Supercapacitor Performances. *ACS Applied Materials & Interfaces* **12**, 18692-18704, doi:10.1021/acsami.0c01655 (2020).

41 Kumar, P. *et al.* Melanin-based flexible supercapacitors. *Journal of Materials Chemistry C* **4**, 9516-9525, doi:10.1039/C6TC03739A (2016).

42 Gouda, A., Soavi, F. & Santato, C. Eumelanin electrodes in buffered aqueous media at different pH values. *Electrochimica Acta* **347**, 136250, doi:<https://doi.org/10.1016/j.electacta.2020.136250> (2020).

43 Xu, R., Gouda, A., Caso, M. F., Soavi, F. & Santato, C. Melanin: A Greener Route To Enhance Energy Storage under Solar Light. *ACS Omega* **4**, 12244-12251, doi:10.1021/acsomega.9b01039 (2019).

44 Guo, W. *et al.* Synthetic melanin facilitates MnO supercapacitors with high specific capacitance and wide operation potential window. *Polymer* **235**, 124276, doi:<https://doi.org/10.1016/j.polymer.2021.124276> (2021).

45 Gouda, A., Manioudakis, J., Naccache, R., Soavi, F. & Santato, C. 3D Network of Sepia Melanin and N- and, S-Doped Graphitic Carbon Quantum Dots for Sustainable Electrochemical Capacitors. *Advanced Sustainable Systems* **n/a**, 2100152, doi:<https://doi.org/10.1002/adsu.202100152>.

46 Hu, L. *et al.* Enhanced electrochemical performance and high voltage window for supercapacitors based on fabric electrodes derived from tannin-Fe3+ complexes. *Synthetic Metals* **269**, 116566, doi:<https://doi.org/10.1016/j.synthmet.2020.116566> (2020).

47 Sanchez-Sanchez, A. *et al.* Excellent electrochemical performances of nanocast ordered mesoporous carbons based on tannin-related polyphenols as supercapacitor electrodes. *Journal of Power Sources* **344**, 15-24, doi:<https://doi.org/10.1016/j.jpowsour.2017.01.099> (2017).

48 Hu, L. *et al.* A scalable strategy for carbon derived from complex six-membered ring-like tannin on glass fiber for 1D/2D flexible all solid state supercapacitors. *Journal of Electroanalytical Chemistry* **856**, 113693, doi:<https://doi.org/10.1016/j.jelechem.2019.113693> (2020).

49 Xu, L. *et al.* Tannic Acid-Decorated Spongy Graphene for Flexible and High Performance Supercapacitors. *Journal of The Electrochemical Society* **165**, A1706-A1712, doi:10.1149/2.0871809jes (2018).

50 Zhao, X., Gnanaseelan, M., Jehnichen, D., Simon, F. & Pionteck, J. Green and facile synthesis of polyaniline/tannic acid/rGO composites for supercapacitor purpose. *Journal of Materials Science* **54**, 10809-10824, doi:10.1007/s10853-019-03654-x (2019).

51 Castro-Gutiérrez, J. *et al.* High-Rate Capability of Supercapacitors Based on Tannin-Derived Ordered Mesoporous Carbons. *ACS Sustainable Chemistry & Engineering* **7**, 17627-17635, doi:10.1021/acssuschemeng.9b03407 (2019).

52 Mukhopadhyay, A. *et al.* Heavy Metal-Free Tannin from Bark for Sustainable Energy Storage. *Nano Lett.* **17**, 7897-7907, doi:10.1021/acs.nanolett.7b04242 (2017).

53 Castro-Gutiérrez, J. *et al.* Model carbon materials derived from tannin to assess the importance of pore connectivity in supercapacitors. *Renewable and Sustainable Energy Reviews* **151**, 111600, doi:<https://doi.org/10.1016/j.rser.2021.111600> (2021).

54 Pérez-Rodríguez, S. *et al.* Upgrading of pine tannin biochars as electrochemical capacitor electrodes. *Journal of colloid and interface science* **601**, 863-876, doi:<https://doi.org/10.1016/j.jcis.2021.05.162> (2021).

55 Jha, S. *et al.* Design and Synthesis of Lignin-Based Flexible Supercapacitors. *ACS Sustainable Chemistry & Engineering* **8**, 498-511, doi:10.1021/acssuschemeng.9b05880 (2020).

56 Jha, S. *et al.* NiWO4 nanoparticle decorated lignin as electrodes for asymmetric flexible supercapacitors. *Journal of Materials Chemistry C* **8**, 3418-3430, doi:10.1039/C9TC05811G (2020).

57 Saha, D. *et al.* Studies on Supercapacitor Electrode Material from Activated Lignin-Derived Mesoporous Carbon. *Langmuir* **30**, 900-910, doi:10.1021/la404112m (2014).

58 Zhang, W. *et al.* 3 D Hierarchical Porous Carbon for Supercapacitors Prepared from Lignin through a Facile Template-Free Method. *ChemSusChem* **8**, 2114-2122, doi:10.1002/cssc.201403486 (2015).

59 Geng, X. *et al.* Bioinspired Ultrastable Lignin Cathode via Graphene Reconfiguration for Energy Storage. *ACS Sustainable Chemistry & Engineering* **5**, 3553-3561, doi:10.1021/acssuschemeng.7b00322 (2017).

60 Mondal, M., Goswami, D. K. & Bhattacharyya, T. K. Lignocellulose based Bio-waste Materials derived Activated Porous Carbon as Superior Electrode Materials for High-Performance Supercapacitor. *Journal of Energy Storage* **34**, 102229, doi:<https://doi.org/10.1016/j.est.2020.102229> (2021).

61 Rajabathar, J. R. *et al.* Synthesis and characterization of metal chalcogenide modified graphene oxide sandwiched manganese oxide nanofibers on nickel foam electrodes for high performance supercapacitor applications. *Journal of Alloys and Compounds* **850**, 156346, doi:<https://doi.org/10.1016/j.jallcom.2020.156346> (2021).

62 Huang, Z. *et al.* Mn3O4 nanoflakes/rGO composites with moderate pore size and (O=)C-O-Mn bond for enhanced supercapacitor performance. *Journal of Alloys and Compounds* **830**, 154637, doi:<https://doi.org/10.1016/j.jallcom.2020.154637> (2020).

63 Du, W. *et al.* Facile synthesis and superior electrochemical performances of CoNi2S4/graphene nanocomposite suitable for supercapacitor electrodes. *Journal of Materials Chemistry A* **2**, 9613-9619, doi:10.1039/C4TA00414K (2014).

64 Wu, H. *et al.* Highly flexible, foldable and stretchable Ni–Co layered double hydroxide/polyaniline/bacterial cellulose electrodes for high-performance all-solid-state supercapacitors. *Journal of Materials Chemistry A* **6**, 16617-16626, doi:10.1039/C8TA05673K (2018).

65 Liu, C. *et al.* Cellulose-derived carbon-based electrodes with high capacitance for advanced asymmetric supercapacitors. *Journal of Power Sources* **457**, 228056, doi:<https://doi.org/10.1016/j.jpowsour.2020.228056> (2020).

66 Bortamuly, R. *et al.* Titania supported bio-derived activated carbon as an electrode material for high-performance supercapacitors. *Journal of Energy Storage* **42**, 103144, doi:<https://doi.org/10.1016/j.est.2021.103144> (2021).

67 Wang, H. *et al.* Flexible Integrated Electrical Cables Based on Biocomposites for Synchronous Energy Transmission and Storage. *Adv. Funct. Mater.* **26**, 3472-3479, doi:10.1002/adfm.201600014 (2016).

68 Anjos, D. M. *et al.* Pseudocapacitance and performance stability of quinone-coated carbon onions. *Nano Energy* **2**, 702-712, doi:<https://doi.org/10.1016/j.nanoen.2013.08.003> (2013).

69 Hu, P. *et al.* Renewable-emodin-based wearable supercapacitors. *Nanoscale* **9**, 1423-1427, doi:10.1039/C6NR09190C (2017).

70 Boota, M. *et al.* Understanding Functionalization of Titanium Carbide (MXene) with Quinones and Their Pseudocapacitance. *ACS Applied Energy Materials* **3**, 4127-4133, doi:10.1021/acsaem.0c00314 (2020).

71 Ma, F. *et al.* Organic Molecular Electrode with Ultrahigh Rate Capability for Supercapacitors. *Energy & Fuels* **34**, 13079-13088, doi:10.1021/acs.energyfuels.0c02207 (2020).

72 Zeng, J., Wei, L. & Guo, X. Bio-inspired high-performance solid-state supercapacitors with the electrolyte, separator, binder and electrodes entirely from kelp. *Journal of Materials Chemistry A* **5**, 25282-25292, doi:10.1039/C7TA08095F (2017).

73 He, S. & Chen, W. Application of biomass-derived flexible carbon cloth coated with MnO2 nanosheets in supercapacitors. *J. Power Sources* **294**, 150-158, doi:<https://doi.org/10.1016/j.jpowsour.2015.06.051> (2015).

74 Kang, Y. J. *et al.* All-Solid-State Flexible Supercapacitors Fabricated with Bacterial Nanocellulose Papers, Carbon Nanotubes, and Triblock-Copolymer Ion Gels. *ACS Nano* **6**, 6400-6406, doi:10.1021/nn301971r (2012).

75 Wang, H. *et al.* Bacterial Cellulose Nanofiber-Supported Polyaniline Nanocomposites with Flake-Shaped Morphology as Supercapacitor Electrodes. *The Journal of Physical Chemistry C* **116**, 13013-13019, doi:10.1021/jp301099r (2012).

76 Yang, T. *et al.* Hierarchical mesoporous yolk–shell structured carbonaceous nanospheres for high performance electrochemical capacitive energy storage. *Chemical Communications* **51**, 2518-2521, doi:10.1039/C4CC09366F (2015).

77 Liu, S., Yu, T., Wu, Y., Li, W. & Li, B. Evolution of cellulose into flexible conductive green electronics: a smart strategy to fabricate sustainable electrodes for supercapacitors. *RSC Advances* **4**, 34134-34143, doi:10.1039/C4RA07017H (2014).

78 Russell, J. C. *et al.* High-performance organic pseudocapacitors via molecular contortion. *Nature Materials* **20**, 1136-1141, doi:10.1038/s41563-021-00954-z (2021).
