# Supplementary material for: Biosourced quinones for high-performance environmentally benign electrochemical capacitors via interface engineering
Source: Commun Chem. 2022 Aug 20;5:98. doi: 10.1038/s42004-022-00719-y (PMC9814668; doi:10.1038/s42004-022-00719-y)
Supplement: Supplementary file 2 — Description of Additional Supplementary Files [file 42004_2022_719_MOESM2_ESM.docx]

Description of Additional Supplementary Files

**File name:** Supplementary Video 1

**Description:** Contact angle of the aqueous electrolyte on untreated CP

**File name:** Supplementary Video 2

**Description:** Contact angle of the aqueous electrolyte on the treated CP
